# Role of protein interactions in stabilizing canonical DNA features in simulations of DNA in crowded environments

**DOI:** 10.1186/s13628-018-0048-y

**Published:** 2018-12-07

**Authors:** Asli Yildirim, Nathalie Brenner, Robert Sutherland, Michael Feig

**Affiliations:** 1 Department of Chemistry, Michigan State University, East Lansing, MI 48824 USA; 2 Faculty of Mathematics and Natural Sciences, University of Düsseldorf, 40225 Düsseldorf, Germany; 3 Department of Biochemistry & Molecular Biology, Michigan State University, 603 Wilson Road, Room BCH 218, East Lansing, MI 48824 USA

**Keywords:** Molecular dynamics, Protein G, A-DNA, B-DNA, Conformational sampling, Solvent interactions

## Abstract

**Background:**

Cellular environments are highly crowded with biological macromolecules resulting in frequent non-specific interactions. While the effect of such crowding on protein structure and dynamics has been studied extensively, very little is known how cellular crowding affects the conformational sampling of nucleic acids.

**Results:**

The effect of protein crowding on the conformational preferences of DNA (deoxyribonucleic acid) is described from fully atomistic molecular dynamics simulations of systems containing a DNA dodecamer surrounded by protein crowders. From the simulations, it was found that DNA structures prefer to stay in B-like conformations in the presence of the crowders. The preference for B-like conformations results from non-specific interactions of crowder proteins with the DNA sugar-phosphate backbone. Moreover, the simulations suggest that the crowder interactions narrow the conformational sampling to canonical regions of the conformational space.

**Conclusions:**

The overall conclusion is that crowding effects may stabilize the canonical features of DNA that are most important for biological function. The results are complementary to a previous study of DNA in reduced dielectric environments where reduced dielectric environments alone led to a conformational shift towards A-DNA. Such a shift was not observed here suggested that the reduced dielectric response of cellular environments is counteracted by non-specific interactions with protein crowders under in vivo conditions.

**Electronic supplementary material:**

The online version of this article (10.1186/s13628-018-0048-y) contains supplementary material, which is available to authorized users.

## Introduction

Biological cells are highly crowded environments due to the presence of various macromolecules. The macromolecular crowding in cells plays a crucial role in biological processes as it may alter the structure and dynamics of biomolecules [1]. A typical biological cell has a concentration of biomolecules in the range of 300–400 mg/ml [2], corresponding to a macromolecular volume fraction of 20–30% [3]. Such an environment is substantially different from dilute solutions, the frequently considered environment in most biological experiments. Recent studies have begun to consider the effects of cellular crowding and have shed light on its effects on the structure and function of biomolecules [4–9]. Three essential crowding effects have been reported from experiments [10] and simulations [11]: (1) the volume exclusion effect has been suggested to favor more compact conformations based on entropic arguments, thereby generally stabilizing more compact states [12, 13]; (2) non-specific interactions between biomolecules and surrounding protein crowders have led to the destabilization of native states [14–16] as well as reduced diffusion [17]; and (3) altered solvation properties including reduced dynamic and dielectric properties [18] have implied a reduced hydrophobic effect [19, 20].

While much attention so far has been on proteins, nucleic acids are also affected by macromolecular crowding [21, 22]. G-quadruplex DNA structure assumes a parallel-G quadruplex form under crowded environments due to the excluded volume effect as well as alterations in the hydration of DNA [23–25]. Long DNA duplexes undergo a collapsing transition in the presence of polyethylene glycol (PEG) in solution, which can also be explained by the volume exclusion effect favoring states that are more compact [26, 27]. The negatively charged protein bovine serum albumin (BSA) similarly causes a compaction of large DNA molecules due to the volume exclusion effect and repulsive electrostatic interactions [28]. Short DNA duplexes, on the other hand, have been extensively investigated by both experimental techniques and computer simulations in terms of co-solvent, salt effects, and crystallization [29–39]. The DNA duplex is well-known to be most stable in the B-form [40] in aqueous solution and in A-form in environments with depleted water and for certain sequences [41]. Very high concentrations of salt can also induce the B- to A- form transition by bringing the negatively charged phosphate groups of DNA closer [34, 38, 42–44] while the addition of ethanol favors the A-form due to reduced electrostatics [32, 33, 35, 37, 45–47]. More recently, the effect of reduced dielectric environments on DNA as one aspect of cellular crowding was investigated and has also been shown to favor non-canonical A-form structures in implicit solvent simulations [20]. On the other hand, a study based on a coarse-grained model has suggested that even in the presence of significant crowding there may be a solvent-rich region around DNA that is depleted in crowder molecules, which was found to have an impact on the kinetics of proteins diffusing along DNA [48]. However, to the extent that crowder proteins do interact non-specifically with DNA, the effect of explicit protein crowder molecules on DNA duplex structures is not well understood.

Here, we describe fully atomistic molecular dynamics (MD) simulations of DNA dodecamers in the presence of explicit protein crowders in order to investigate how DNA structure and stability may be affected under such conditions. We find a general tendency of the DNA to favor the B-form in crowded environments, which is in contrast to the shift towards A-form DNA observed in the simpler reduced dielectric environments [20]. The stabilization of B-DNA appears to be due to non-specific protein-DNA interactions. We also observe, some alterations in the hydration structure and ion distributions around DNA under crowded conditions. The results are described in detail and discussed in the following after outlining the computational methods used in this study.

## Methods

MD simulations of Drew-Dickerson ((CGCGAATTCGCG)_2_) and GC-rich (CGCCCCGCGGGCG)_2_) dodecamers in crowded protein environments were carried out using the CHARMM (Chemistry at Harvard Molecular Mechanics) program package (version 41a1) [49] with the CHARMM36 force field [50, 51]. The initial Drew-Dickerson dodecamer structure was obtained from the X-ray structure (PDB: 1BNA) [52], and the initial GC-rich dodecamer structure was obtained by mutating the base sequence in the X-ray structure of the Drew-Dickerson dodecamer using the MMTSB (Molecular Modeling Tools in Structural Biology) Tool Set [53]. In experiment, the Drew-Dickerson dodecamer is very stable in B-form, in the crystal as well as in solution [52]. The crystal structure of the GC-rich dodecamer is in A-form [54], but there is less known about its conformation in solution. Generally there is little evidence for A-DNA conformations in solution unless the salt concentration is much higher than typical physiological conditions [34, 38, 42–44] and/or when co-solvents such as ethanol are present in significant fractions [32, 33, 35, 37, 45–47]. Therefore, we setup both systems in B-form as the likely conformation of both sequences in dilute aqueous solvent. The choice of sequences and initial structures also allows a direct comparison with our previous continuum dielectric study [20].

For each dodecamer, a dilute system without crowders (0% crowder fraction) and three systems with different protein crowder volume fractions (20, 30, 40%) were prepared. Protein G (PDB: 1PGB) [55] was selected as the crowder protein due to its small size and stability in computer simulations [15]. We used neutral protein G models molecules introduced in previous work [56], where D36, D40, E19 and E42 are protonated. In the previous study, both, the charged and neutralized variants of protein G were studied under crowded conditions similar to the systems studied here but without DNA and both were found to be stable in simulations [56]. Protein G is not known to specifically interact with DNA and we chose the net-neutral form to reduce electrostatic interactions with the highly charged DNA to focus on more general crowding effects while still maintaining protein-like crowders. The crowded systems (20, 30, 40%) consisted of one dodecamer and 8 protein G molecules, whereas the dilute systems only contained one dodecamer. Simulation box sizes were varied between 53.2–61.3 Å to obtain the abovementioned crowder volume fractions. The box sizes were varied instead of the number of protein copies to achieve exactly the target crowder fractions and minimize computational costs at the higher concentrations as in previous work [15, 57]. Simulation conditions of the systems are given in Table 1. There is no experimental evidence for a specific DNA-protein G complex that is stable over long time and consequently the system is assumed to be fully dynamic in the liquid state with molecular interactions varying transiently. To avoid biasing towards any specific initial protein G-DNA interaction, the initial crowded systems were set up by randomly rotating and placing the DNA dodecamer and the crowder proteins in the simulation box using a protocol developed previously [58]. Different replicates of each system had different initial orientations and placements of the DNA and the surrounding crowders. All systems were solvated with explicit TIP3P (three-site transferable intermolecular potential) [59] water molecules. To neutralize the DNA dodecamer, 22 sodium ions were added to the systems. In order to keep the ion molality of all systems the same, 6 and 12 additional pairs of sodium and chloride ions were added to 30% and 0/20% systems, respectively. Therefore, all systems had 0.45 mol/kg ion molality.

**Table 1:** Overview of Simulations

DNA Sequence	Size (Å)	Protein Vol (%)	Protein Conc. (g/L)	Ion Molarity (M)	Ion Molality (mol/kg)	Number of atoms	Simulation Length (μs)	Number of replicates
Drew-Dickerson	54.62	0	0.00	0.45	0.45	16,685-16,720	1	3
Drew-Dickerson	61.02	20	362.49	0.32	0.45	23,958-24,087	1	5
Drew-Dickerson	57.90	30	424.31	0.29	0.45	20,384-20,483	1	5
Drew-Dickerson	53.21	40	546.68	0.24	0.45	15,824-16,019	1	5
GC-rich	56.26	0	0.00	0.43	0.45	18,028-18,059	1	3
GC-rich	61.31	20	357.37	0.32	0.45	24,125-24,445	1	5
GC-rich	57.74	30	427.84	0.29	0.45	20,353-20,377	1	5
GC-rich	53.18	40	547.61	0.24	0.45	15,499-15,904	1	5

The initial systems were minimized for 1000 steps using the adopted bases Newton Raphson (ABNR) algorithm and were subsequently heated by running simulations without using any restraints at 50 K, 100 K, 150 K, 200 K, 250 K for 4 ps and at 298 K for 10 ps. Productions runs were carried out at 298 K in the NVT ensemble for 1 μs with a 2 fs time step. The SHAKE algorithm [60] was used to constrain bond lengths involving hydrogen atoms. Temperature control was obtained by a Langevin thermostat with a 0.01 ps^− 1^ friction coefficient. Lennard-Jones and direct electrostatic interactions were cut off at 12 Å with a switching function becoming effective at 10 Å. Electrostatic interactions were calculated from particle-mesh Ewald [61] summation using 1 Å grid spacing. All simulations were performed using periodic boundary conditions. For the crowded systems, five independent simulations were carried out starting from different initial orientations. For the dilute systems without protein crowders, simulations were replicated three times starting from different initial velocities for the atoms.

The analysis of the helicoidal and backbone parameters of the dodecamers (see Additional file 1: Figure S1) were performed by using the 3DNA program package [62]. The reported values are averages over snapshots. Radial distribution functions and 3D volume densities were analyzed by using in-house scripts. All the other analysis was carried out using the MMTSB Tool Set [53] in combination with CHARMM [49]. Clustering analysis was performed by applying the k-means clustering algorithm by using the *kclust* program in MMTSB [53]. For each dodecamer, all snapshots from the simulations with different protein concentrations were aggregated and clustered by using a 3 Å clustering radius. Only the last 700 ns of the simulations were analyzed because of larger variations in the helicoidal parameters during the first 300 ns (see Additional file 1: Figure S2). Only the inner eight base-pairs were taken into consideration to ignore structural distortions due to base fraying. VMD (visual molecular dynamics) [63] and PyMOL [64] were used for visualization.

## Results

Microsecond-scale molecular dynamics simulations of DNA dodecamers with and without protein crowders were carried to study the effect of crowding on DNA structure. We focused our analysis on helical properties including base geometries, groove widths and DNA bending, backbone torsions, interactions with crowder proteins, correlations between protein contacts and helical properties, and water and ion distributions around DNA.

### Helical properties

**Fig. 1:**
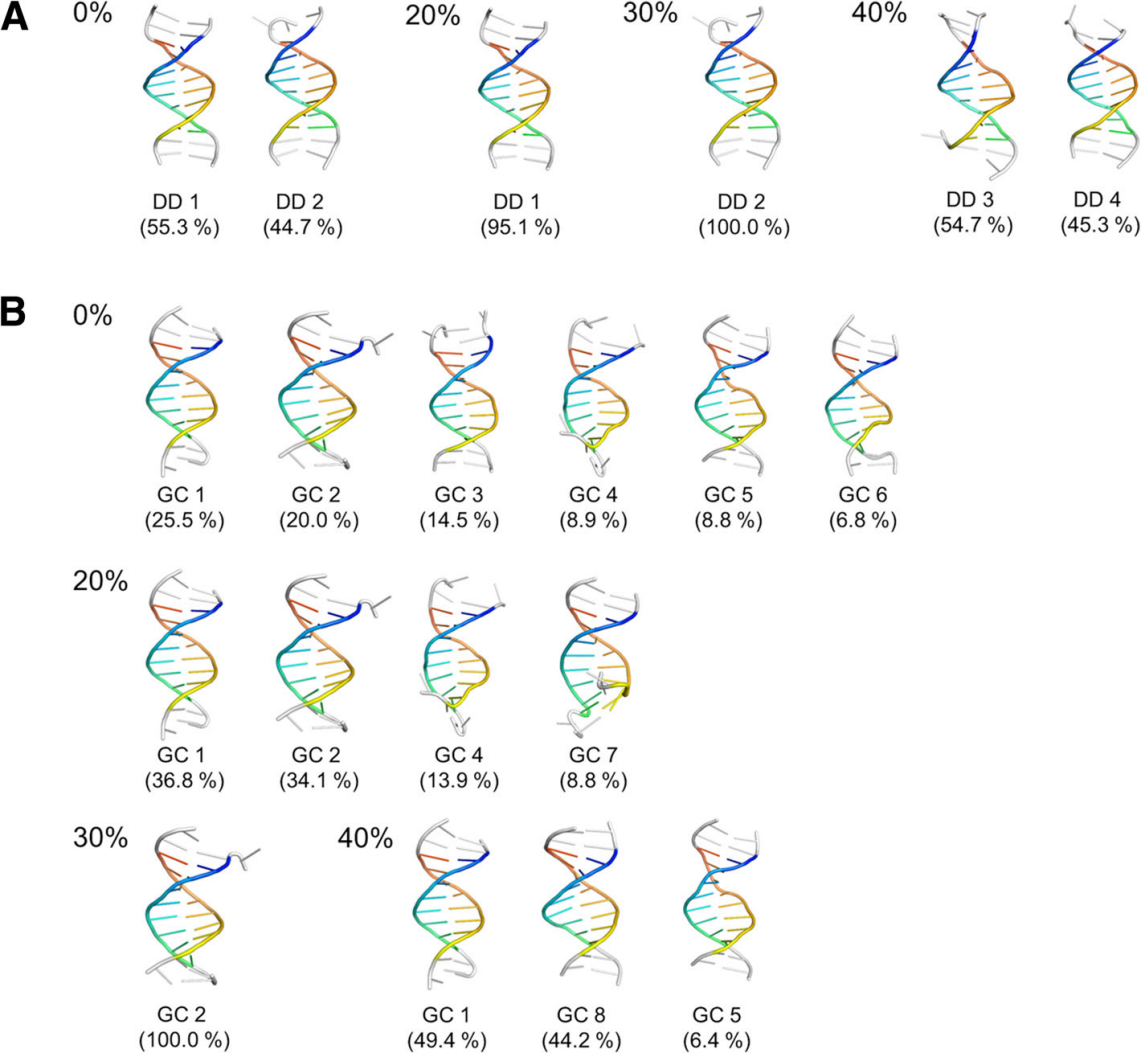
Representative conformations from clustering simulation snapshots for the Drew-Dickerson (**a**) and GC-rich (**b**) dodecamers at 0, 20, 30 and 40% protein concentrations. The structures are the structures in each cluster closest to the closest center based on RMSD. Cluster populations are given in parentheses

Snapshots from the simulations were clustered to identify major conformations. Representative structures for each of the major clusters (with more than 5% population) are depicted in Fig. 1. Generally, the helices stayed intact with base fraying at the termini, which is common in simulations of short DNA fragments [65]. The structures generally resemble B-DNA structures for both sequences. Average root mean square deviation (RMSD) values of different clusters from the initial canonical B-DNA structures vary between 1.4 and 2.0 Å for the Drew-Dickerson dodecamer and between 1.6 to 2.6 Å except for one cluster with an RMSD of 3.7 Å for the GC-rich dodecamer (see Additional file 1: Table S1). There is no clear pattern of increasing or decreasing RMSD values for the clusters most populated at different crowder concentrations.

Averages over all base-pairs excluding the first and last two terminal base-pairs with errors given in parentheses based on the variations in the independent simulations. Canonical values were averaged over the A-form structures 3V9D, 3QK4, 2B1B, 1ZEX, 1ZEY, 1ZF1, 1ZF8, 1ZF9, 1ZFA and the B-form structures 2M2C, 4AGZ, 4H0, 4AH1, 3 U05, 3 U08, 1VTJ,3U2N, 3OIE, 3BSE.

see Table 2.

**Table 2:** Average Helical Parameters for the Drew-Dickerson Dodecamer

		Canonical		Simulations in crowded environment			
	X-ray (1BNA)	A-DNA	B-DNA	0%	20%	30%	40%
Slide (Å)	0.07 (*0.20*)	−1.62 (*0.06*)	0.16 (*0.08*)	0.26 (*0.04*)	0.25 (*0.06*)	0.21 (*0.04*)	0.25 (*0.05*)
Twist (deg)	34.22 (*2.13*)	30.34 (*0.58*)	34.70 (*0.70*)	33.42 (*0.18*)	34.06 (*0.25*)	33.96 (*0.38*)	33.43 (*0.31*)
X-displacement (Å)	−0.23 (*0.20*)	−4.50 (*0.18*)	−0.20 (*0.13*)	− 0.59 (*0.10*)	− 0.60 (*0.14*)	− 0.72 (*0.11*)	− 0.79 (*0.12*)
Helical rise (Å)	3.29 (*0.05*)	2.68 (*0.08*)	3.25 (*0.02*)	3.24 (*0.00*)	3.27 (*0.01*)	3.25 (*0.02*)	3.27 (*0.02*)
Inclination (deg)	4.02 (*2.73*)	17.78 (*1.56*)	4.34 (*0.77*)	10.87 (*0.22*)	10.62 (*0.44*)	11.15 (*0.59*)	12.59 (*0.77*)
z_p_ (Å)	−0.23 (*0.07*)	2.06 (*0.07*)	−0.33 (*0.04*)	− 0.07 (*0.03*)	0.12 (*0.05*)	0.14 (*0.05*)	0.22 (*0.04*)
Minor groove (Å)	10.32 (*0.46*)	15.72 (*0.12*)	10.77 (*0.12*)	13.50 (*0.05*)	13.42 (*0.18*)	13.08 (*0.14*)	13.73 (*0.19*)
Major groove (Å)	17.34 (*0.33*)	12.94 (*0.39*)	17.14 (*0.12*)	16.47 (*0.11*)	16.54 (*0.10*)	16.39 (*0.15*)	16.31 (*0.12*)

Helicoidal parameters for both, Drew-Dickerson and GC-rich dodecamers were averaged from the simulations. They are summarized in Tables 2 and 3, respectively. Helicoidal parameters for crystal structures of the respective dodecamers as well as canonical A- and B-forms of DNA, averaged over ten A- and B-form crystal structures each, are provided for comparison. Average properties for each of the clusters shown in Fig. 1 are given in Additional file 1: Tables S1 and S2. The more detailed analysis of the base geometries also indicates that both dodecamers remained close to B-DNA. The Drew-Dickerson dodecamer also remained reasonably close to the respective crystal structure (1BNA), but there are larger deviations between the simulation results and the crystal structure of the GC-rich dodecamer. The crystal structure for the GC-rich dodecamer is predominantly in A-form, presumably as a result of salt concentrations above 1 M and/or the crystal environment [54]. As mentioned above, although the crystal structure of the GC-rich dodecamer has been reported in A-form, there is no evidence that this sequence (or any other DNA sequence) assumes an A-DNA conformation in solution at sub-molar salt concentrations and in the absence of co-solvents. Therefore we expected the GC-dodecamer to remain in B-form. In the presence of the protein crowders, the helical parameters generally did not change much. We found increased X-displacement (*p*-values: 0.91 (Drew-Dickerson 20%), 0.16 (Drew-Dickerson 30%), 0.05 (Drew-Dickerson 40%), 0.05 (GC-rich 20%), 0.04 (GC-rich 30%), 0.07 (GC-rich 40%)) and base inclination (p-values: 0.33 (Drew-Dickerson 20%), 0.38 (Drew-Dickerson 30%), 0.01 (Drew-Dickerson 40%), 0.05 (GC-rich 20%), 0.01 (GC-rich 30%), 0.08 (GC-rich 40%)) for both dodecamers as a function of crowding. The increased x-displacement and base inclination point towards A-DNA but the values upon crowding still remained much closer to canonical B-DNA than A-DNA.

**Table 3:** Average Helical Parameters for the GC-rich Dodecamer

		Canonical		Simulations in crowded environment			
	X-ray (399D)	A-DNA	B-DNA	0%	20%	30%	40%
Slide (Å)	−1.71 (*0.16*)	−1.62 (*0.06*)	0.16 (*0.08*)	0.32 (*0.19*)	0.02 (*0.10*)	0.02 (*0.10*)	0.00 (*0.07*)
Twist (deg)	29.59 (*1.34*)	30.34 (*0.58*)	34.70 (*0.70*)	32.47 (*0.85*)	32.93 (*0.42*)	33.34 (*0.41*)	33.11 (*0.36*)
X-displacement (Å)	−5.01 (*0.41*)	−4.50 (*0.18*)	−0.20 (*0.13*)	−0.46 (*0.28*)	−1.03 (*0.20*)	− 1.09 (*0.22*)	−1.10 (*0.16*)
Helical rise (Å)	2.66 (*0.22*)	2.68 (*0.08*)	3.25 (*0.02*)	3.26 (0*.03*)	3.23 (*0.04*)	3.26 (*0.02*)	3.27 (*0.02*)
Inclination (deg)	20.71 (*4.33*)	17.78 (*1.56*)	4.34 (*0.77*)	10.01 (*0.33*)	10.70 (*0.38*)	11.34 (*0.49*)	11.34 (*1.19*)
z_p_ (Å)	1.56 (*0.35*)	2.06 (*0.07*)	−0.33 (*0.04*)	−0.21 (*0.12*)	−0.20 (*0.09*)	− 0.23 (*0.07*)	−0.18 (*0.05*)
Minor groove (Å)	16.22 (*0.47*)	15.72 (*0.12*)	10.77 (*0.12*)	14.91 (*0.23*)	14.54 (*0.08*)	14.52 (*0.10*)	14.76 (*0.21*)
Major groove (Å)	13.14 (*2.63*)	12.94 (*0.39*)	17.14 (*0.12*)	16.26 (*0.16*)	16.53 (*0.13*)	16.22 (*0.14*)	16.27 (*0.23*)

We further analyzed the displacement of phosphorus atoms relative to the horizontal plane passing between base-pairs in a base-pair step (z_p_) and major/minor grooves (Tables 2 & 3). The z_p_ parameter is very different between the two forms of DNA. While B-DNA has values near − 0.3 Å, the parameter is mostly larger than 2.0 Å for A-DNA. This parameter does not show a trend upon crowding for the GC-rich dodecamer, while the Drew-Dickerson dodecamer had larger values in crowded environments (*p*-values: 0.0011 (20%), 0.0007 (30%), 0.0001 (40%)). Again this indicates a slight tendency towards A-DNA geometries while still remaining much closer to canonical B-DNA values. Minor and major groove widths also did not change significantly upon crowding, but we note that minor groove widths were generally overestimated compared to canonical B-DNA values. This is a general feature of the CHARMM force field that was used here [66]. Finally, we analyzed the helical bending angles (see Additional file 1: Table S3) which also did not show a significant change upon crowding.

### Sugar conformations and backbone torsions

**Fig. 2:**
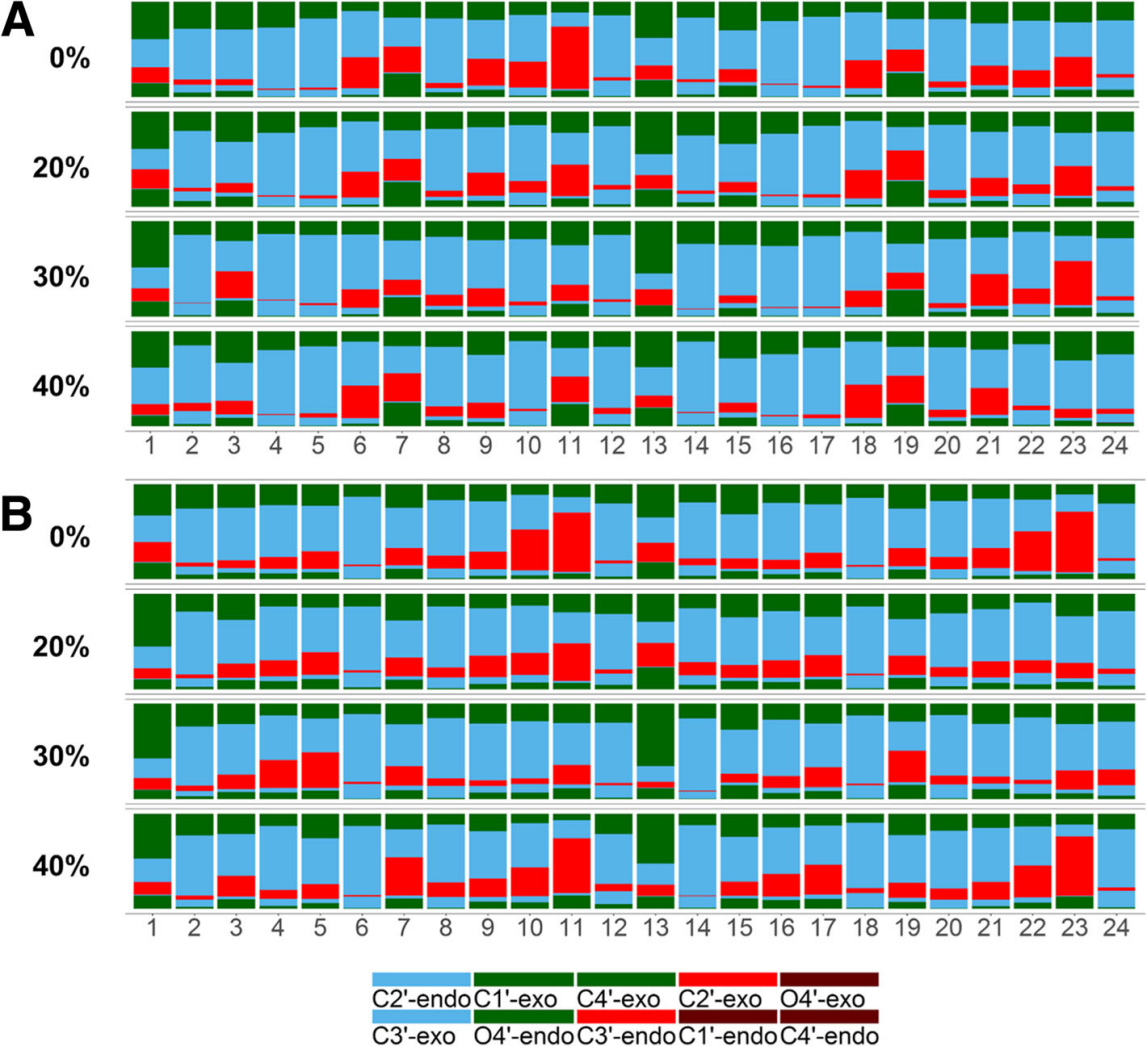
Sugar pucker conformations for each base of the Drew-Dickerson dodecamer (**a**) and the GC-rich dodecamer (**b**) from simulations at different protein concentrations

A key feature of nucleic acid backbone is the ribose pucker conformation. A-form DNA is known to prefer C3^’^-endo and C2^’^-exo conformations whereas B-form DNA is characterized by C3^’^-exo and C2^’^-endo conformations. As shown in Fig. 2, the sugars of both dodecamers generally remained in C3^’^-exo and C2^’^-endo conformations. As expected, C3^’^-endo and C2^’^-exo sugar conformations are more prominent for the GC-rich dodecamer (see Fig. 2b). Again, there is no major change upon crowding, but in the GC-rich dodecamer, sugars shift slightly to C3^’^-exo and C2^’^-endo sugar conformations up to 30% crowding, but then revert back to more A-form conformations at 40% crowder concentrations.

**Fig. 3:**
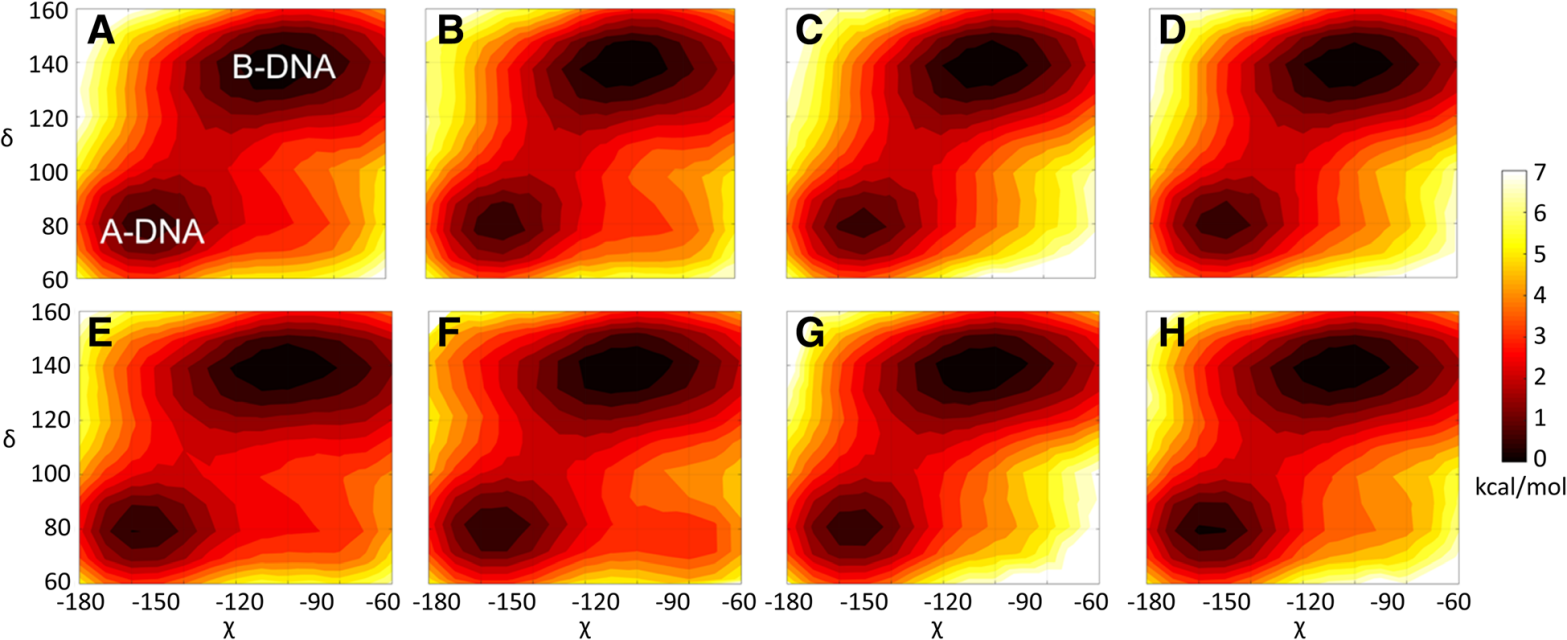
Potentials of mean force (kcal/mol) as a function of δ and χ backbone angles (see Additional file 1: Figure S1) for the Drew-Dickerson dodecamer at 0% (**a**), 20% (**b**), 30% (**c**) and 40% (**d**) protein concentrations, and for the GC-rich dodecamer at 0% (**e**), 20% (**f**), 30% (**g**) and 40% (**h**) protein concentrations from the simulations. See Additional file 1: Table S5 for uncertainties

We further analyzed torsion angles along the phosphate backbone. χ and δ angles are the most distinctive backbone angles to distinguish between A- and B- form DNA. We constructed potentials of mean force (PMF) as a function of δ and χ from the simulations (Fig. 3). The separation between A- and B-DNA torsion angles is readily apparent. Consistent with the ribose puckers and helical geometries, there is more sampling of B-DNA torsion angles for both dodecamers. While there is little change in the sampling of the major A- and B-form, the presence of crowders appears to affect the sampling of minor conformations with A-like δ values around 80 degrees and B-like χ values around − 100 degrees. Sampling in this region is significantly reduced in both dodecamers upon crowding (see Fig. 3). This region corresponds to a conformation where bases stay in the same orientation relative to the sugar as in B-form, but they are slightly more exposed to the environment, and apparently, this conformation is largely prevented by crowder proteins. The sampling of ε and ζ torsion angles distinguishes between BI/BII forms. A similar trend is observed where crowding reduces the sampling of minor states outside the major BI/BII basins (see Additional file 1: Figure S3). Based on this analysis, it appears that one effect of protein crowders may be to focus the sampling of DNA conformations on the major conformations.

### Protein crowder conformations

In previous simulation studies involving protein G under crowded conditions, the protein G conformations remained close to the experimental structure and were not affected strongly by the concentrated environment [15]. In the systems studied here, protein G also remains highly stable and close to the experimental structure (see Additional file 1: Figure S4). The overall average Cα RMSD value is 0.91 Å with a standard deviation of 0.24 Å between individual protein G molecules and the experimental structure (PDB ID: 3GB1 [55, 67]). The average radius of gyration is 10.76 Å with a standard deviation of 0.1 A, compared to a value of 10.65 Å for the experimental structure. A few conformations deviated slightly further from the native (as much as 2.5 Å Cα RMSD) and with slightly increased radii of gyration, especially at the highest crowder concentration (Additional file 1: Figure S4B). Further analysis via clustering revealed minor substates with slightly increased RMSD values that correlate with closer contacts to the DNA (see Additional file 1: Table S4). This suggests that the conformational sampling of protein G may be affected slightly when interacting with the DNA. Almost all of the variations are in the flexible loop involving residues 9 to 13 (see Additional file 1: Figure S4D) where root mean square fluctuations (RMSF) are largest (see Additional file 1: Figure S4C).

### DNA-protein interactions

**Fig. 4:**
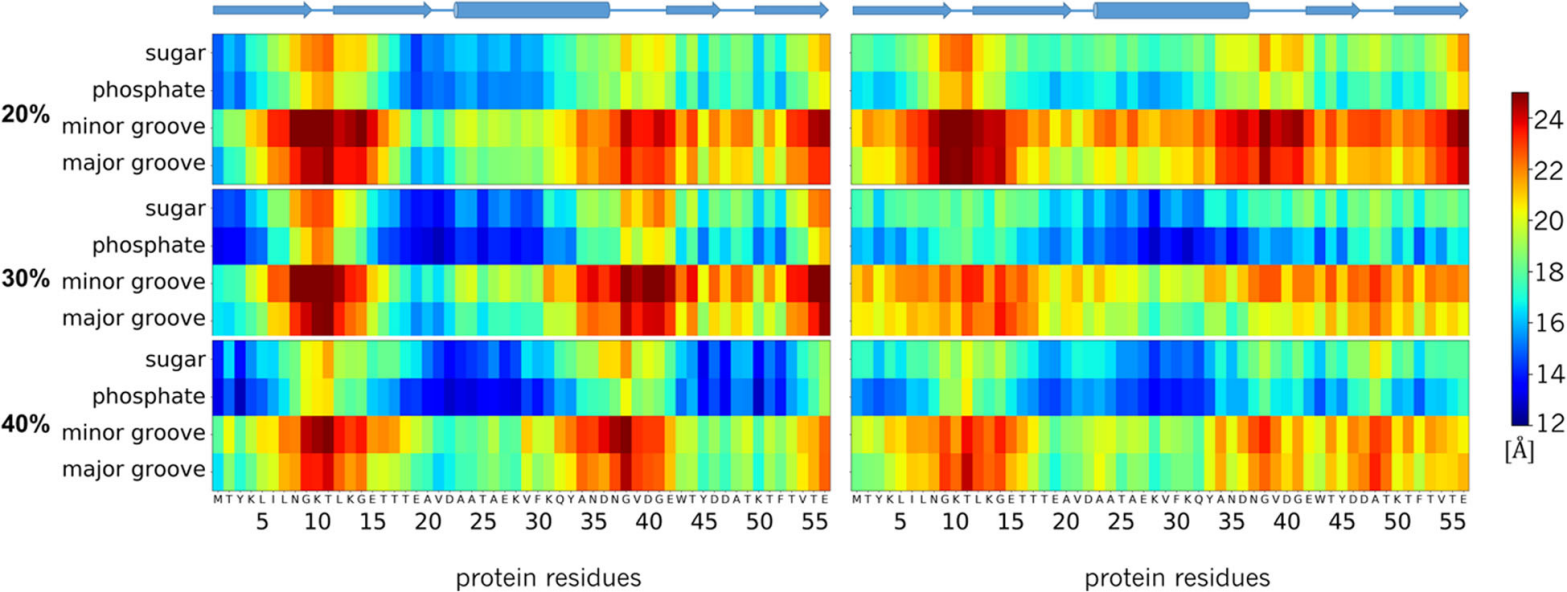
Average minimum heavy atom distances between crowder protein residues and DNA major groove, minor groove, sugar and phosphate backbone for the Drew-Dickerson dodecamer (left) and the GC-rich dodecamer (right) at different protein concentrations. The secondary structure of protein G is indicated on top for reference

**Fig. 5:**
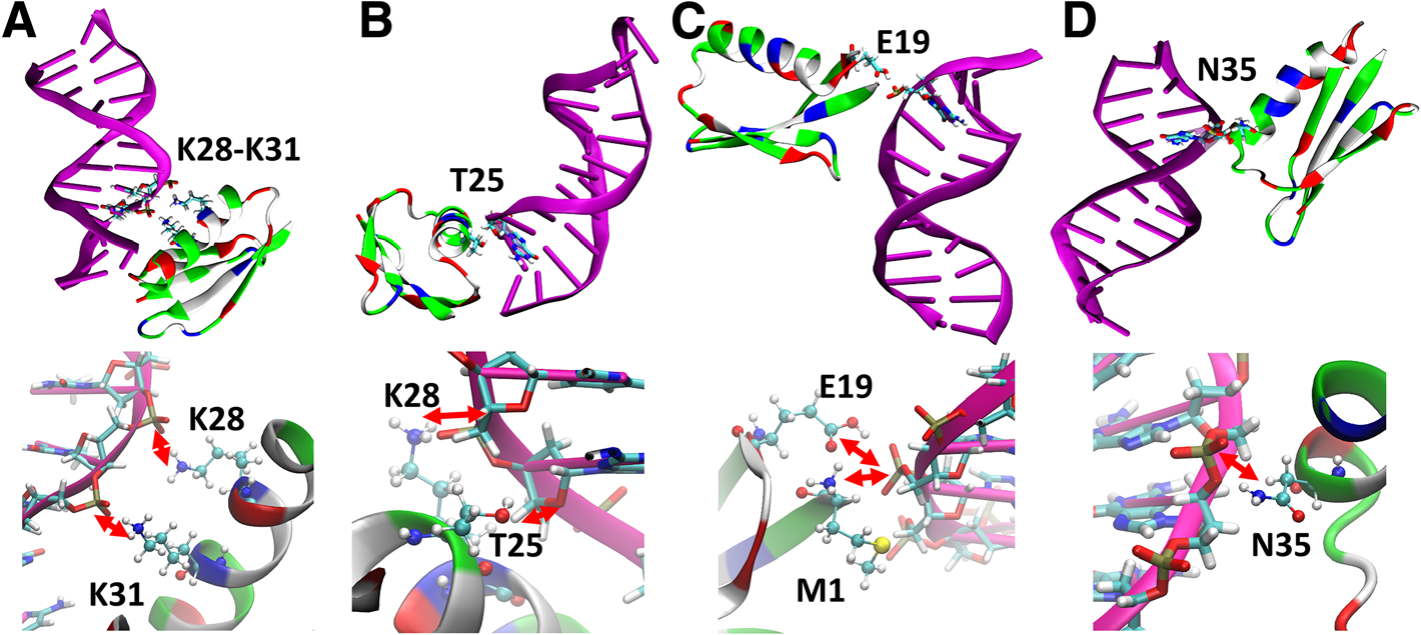
Representative structures showing interactions between crowder proteins and the Drew-Dickerson dodecamer (**a**, **b**) and the GC-rich DNA (**c**, **d**). Specific interactions are shown between lysines and the DNA phosphate groups (A), between a threonine residue and a ribose sugar (**b**), between protonated glutamate and a phosphate (**c**), and between asparagine and the phosphate (**d**). Interacting residues are shown in licorice (DNA) and ball-and-stick (protein) representation. The chosen structures have minimal distances between the DNA and the protein

Protein G is not known to interact specifically with DNA but under highly crowded conditions, interactions are unavoidable. Figure 4 shows where contacts between protein G and DNA occur based on minimum distances between the major/minor grooves and sugar/phosphate groups of the DNA with different residues of protein G. More detailed contact analysis between individual base-pairs and protein G residues is shown in Additional file 1: Figures S5 and S6 for the Drew-Dickerson and GC-rich dodecamers, respectively. Most of the contacts are between the DNA sugar-phosphate backbone and protein residues 15–30, mostly in the α-helix of protein G, as well as residues at the N-terminus and near the C-terminus. Contacts involving the DNA grooves, a typical mode of interaction for DNA-binding proteins were not common with protein G. The interactions partially involve electrostatic attraction between the DNA phosphate and certain lysine residues (K4, K28, K31, and K50), but sugar oxygens O3’ and O4’ as well as phosphate oxygens also form hydrogen bonds with other polar protein residues. Representative snapshots of protein G-DNA interactions are shown in Fig. 5. As would be expected, the contacts between the proteins and DNA increase with crowder concentration and crowding seems to increase sugar-phosphate-protein contacts more for the Drew-Dickerson dodecamer than for the GC-rich dodecamer.

### Correlations between DNA-protein contacts and DNA helix properties

**Fig. 6:**
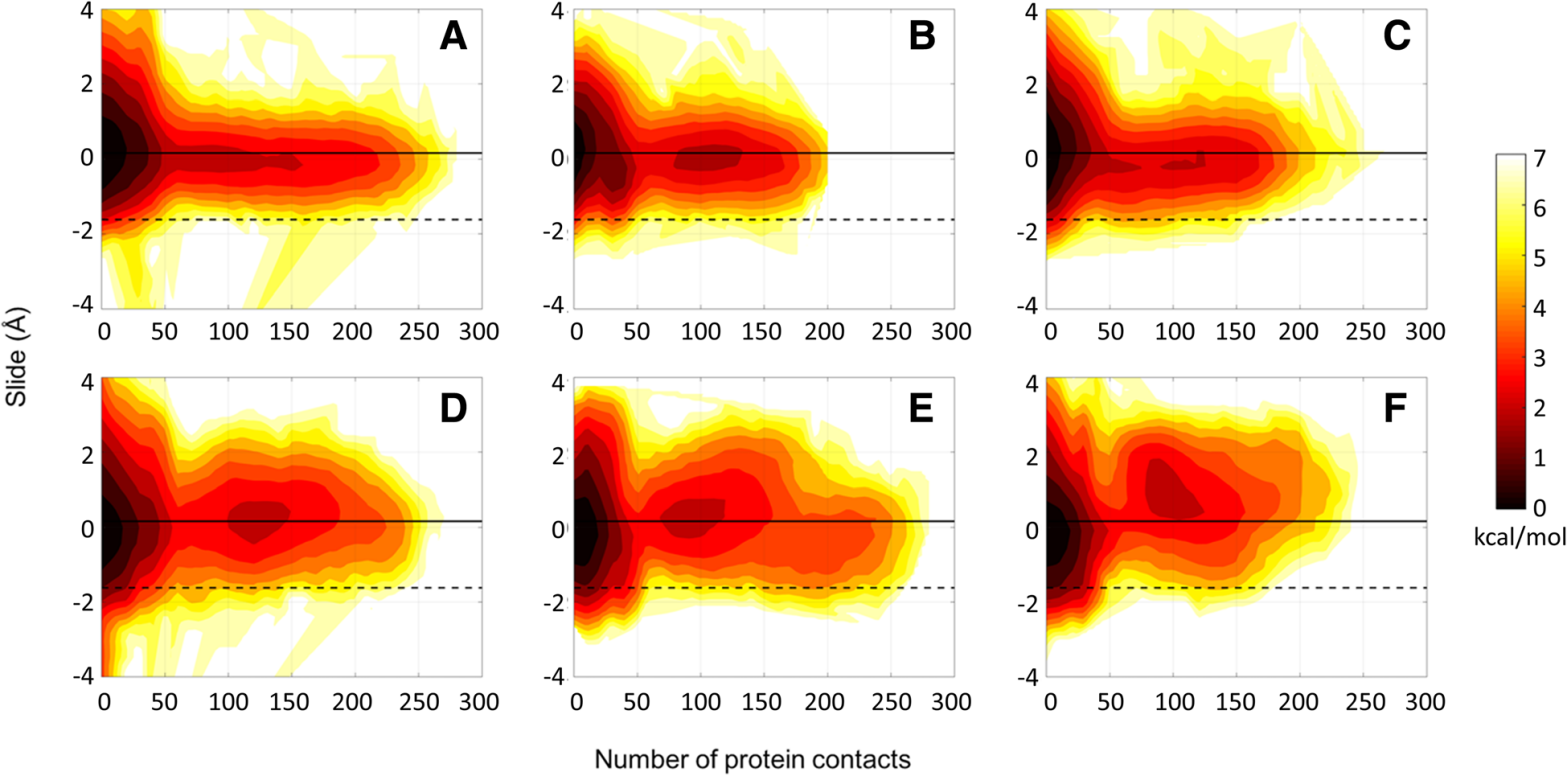
Potentials of mean force (kcal/mol) as a function of slide (see Additional file 1: Figure S1) and number of protein contacts for the Drew-Dickerson dodecamer at 20% (**a**), 30% (**b**), 40% (**c**) protein concentrations, and for the GC-rich dodecamer at 20% (**d**), 30% (**e**), 40% (**f**) protein concentrations from the simulations. A contact is defined when the minimum distance between the heavy atoms of crowder proteins and DNA phosphate groups is less than 5 Å. Solid and dashed lines indicate the slide and x-displacement values for canonical B- and A-forms, respectively. See Additional file 1: Table S5 for uncertainties

**Fig. 7:**
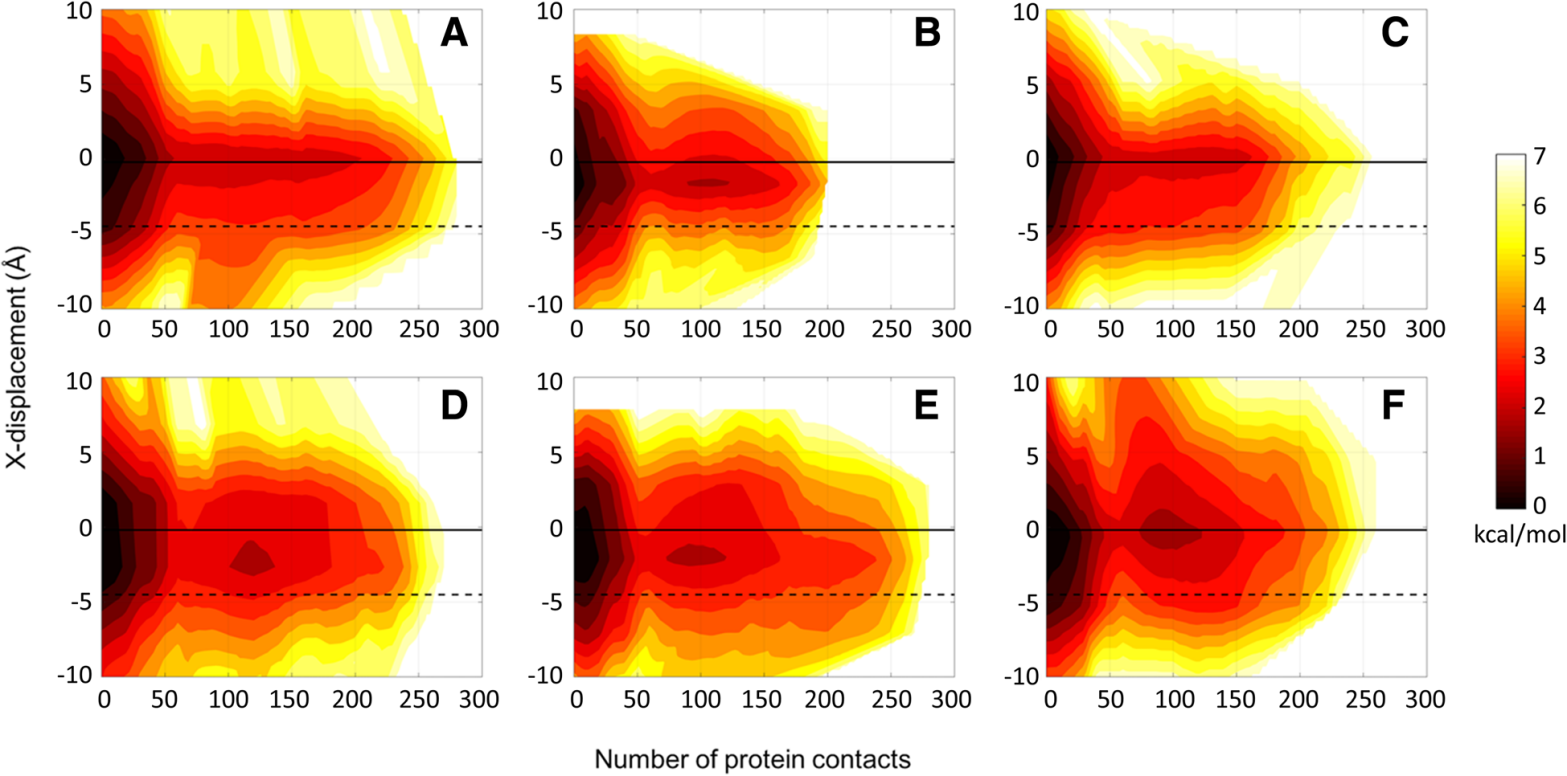
Potentials of mean force (kcal/mol) as a function of x-displacement (see Additional file 1: Figure S1) and number of protein contacts for the Drew-Dickerson dodecamer at 20% (**a**), 30% (**b**), 40% (**c**) protein concentrations, and for the GC-rich dodecamer at 20% (**d**), 30% (**e**), 40% (**f**) protein concentrations from the simulations. See Additional file 1: Table S5 for uncertainties

**Fig. 8:**
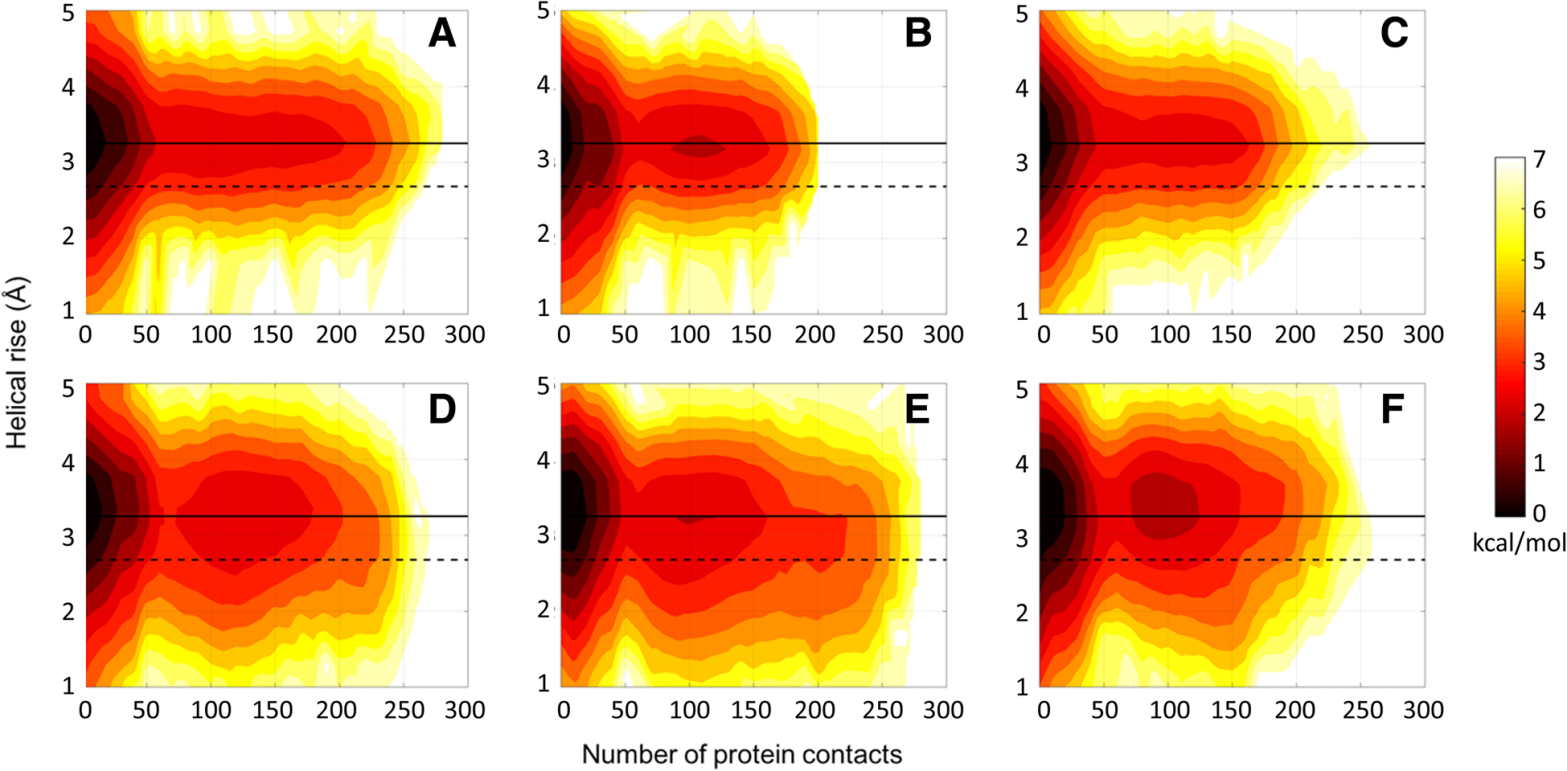
Potentials of mean force (kcal/mol) as a function of helical rise (see Additional file 1: Figure S1) and number of protein contacts for the Drew-Dickerson dodecamer at 20% (**a**), 30% (**b**), 40% (**c**) protein concentrations, and for the GC-rich dodecamer at 20% (**d**), 30% (**e**), 40% (**f**) protein concentrations from the simulations. See Additional file 1: Table S5 for uncertainties

**Fig. 9:**
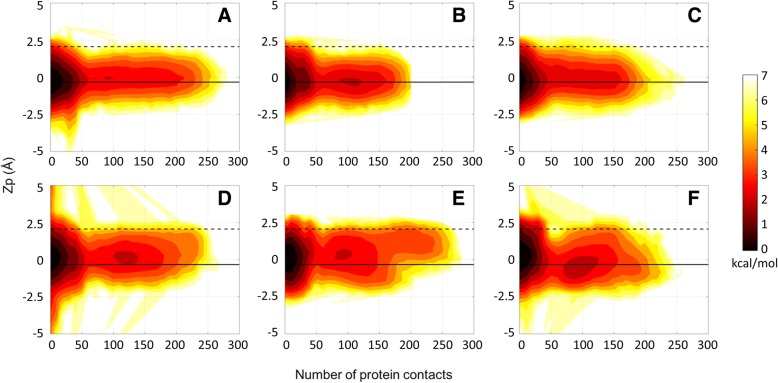
Potentials of mean force (kcal/mol) as a function of Z_p_ (see Additional file 1: Figure S1) and number of protein contacts for the Drew-Dickerson dodecamer at 20% (**a**), 30% (**b**), 40% (**c**) protein concentrations, and for the GC-rich dodecamer at 20% (**d**), 30% (**e**), 40% (**f**) protein concentrations from the simulations. See Additional file 1: Table S5 for uncertainties

To investigate in more detail whether the close contacts of the crowder proteins with the DNA have the potential to perturb DNA structure, we analyzed correlations between DNA-protein contacts and helicoidal properties of DNA as well as backbone torsion and pseudorotation phase angles. First, we examined the effect of close contacts on the helicoidal parameters listed in Tables 2 and 3. We found that a higher number of close protein contacts corresponded to a more narrow range of sampled values for all of the helicoidal parameters (Figs. 6-9, Additional file 1: Figures S7-S10). Among these parameters, slide (Fig. 6), x-displacement (Fig. 7), helical rise (Fig. 8) and z_p_ (Fig. 9) values showed a clear shift towards B-form values with increasing number of contacts. These parameters focus on the displacement of bases along the x- (x-displacement) and y- (slide) axes and of phosphates along the base-pair axis (z_p_). All of the values approach zero with crowding. This suggests that DNA bases and phosphates undergo less displacement as a result of crowding. On the other hand, rotations of base-pairs about helical (twist) or base-pair axes (inclination) do not show a distinct shift towards any canonical values (Additional file 1: Figures S7, S8). Major and minor groove widths do not seem to be affected by contacts except for the GC-rich dodecamer, where there appears to be a clear tendency towards larger minor groove values, i.e. values more similar to A-DNA (Additional file 1: Figures S9, S10).

Similar to the helicoidal parameters, backbone torsion angles also fluctuate in a more narrow range upon crowding (Additional file 1: Figures S11–S17). This suggests that non-specific protein-DNA interactions may limit the conformational fluctuations of the DNA backbone. Particularly, δ and χ angles shift towards B-form values upon higher number of protein contacts, explaining a decrease in the sampling of non-canonical conformations shown in Fig. 3. Finally, pseudorotation angles move to B-form values with protein contacts which lead to C3^’^-exo and C2^’^-endo sugar pucker conformations (Additional file 1: Figure S18).

The results discussed here are most pronounced for the Drew-Dickerson dodecamer. In the GC-rich dodecamer, the fluctuations of helicoidal parameters and backbone angles are reduced less and a tendency to sample A-form values further complicates the picture. Overall, our results suggest that the interactions of protein crowders with DNA sugar/phosphate backbone shown in the previous section result in a stiffer DNA backbone. The stiffer backbone also prevents larger base/base-pair displacements and, therefore, restricts the conformational space of DNA. Although it appears that there is not a specific tendency towards one of the major forms of DNA upon crowding, there is a distinct effect of protein crowders on DNA structure by narrowing the conformational sampling to canonical structures.

### Hydration and ion distributions around DNA

**Fig. 10:**
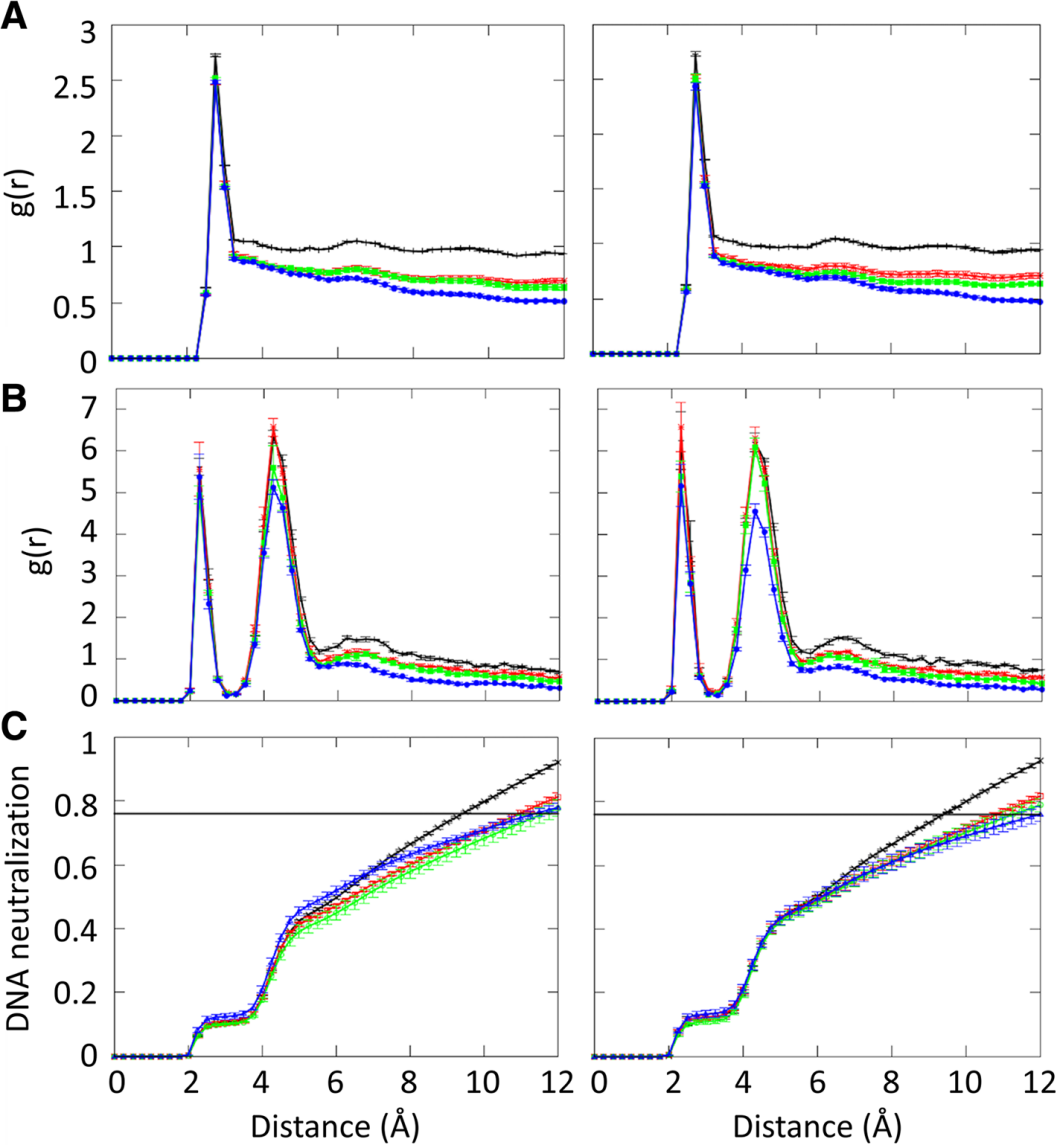
Radial distribution functions for water (**a**), sodium ions (**b**) and DNA neutralization fractions (**c**) as a function of distance from the closest heavy atoms of the Drew-Dickerson dodecamer (left) and the GC-rich dodecamer (right). Line colors indicate different protein concentrations (black: 0%, red: 20%, green: 30%, blue: 40%). The horizontal black line in C indicates the counterion condensation value of 76% of the ions to be condensed on the surface of the DNA. Error bars indicate the calculated standard errors from five independent replicate simulations

Water and ions are integral parts of DNA structures. We analyzed hydration patterns and sodium ion distributions around DNA as a function of crowding. Conditional water radial distribution functions (RDF) were obtained for water oxygen distances to the closest heavy atoms in DNA, normalized by the corresponding accessible volume at each distance and the bulk water density (0.034 Å^− 3^) (see Fig. 10a). The analysis shows that the first hydration shell is almost unaffected by the level of crowding, but the RDF decreases beyond the hydration shell significantly as a function of crowding. This observation is similar to what has been reported previously for the hydration around proteins under crowded environments [18].

Sodium RDFs were calculated in the same way as the water RDFs but normalized by the ion density of the system (0.002 Å^− 3^). There are two peaks in the sodium RDFs corresponding to ions in direct contact with the DNA (around 2.5 Å and largely in the minor groove) and ions interacting with the DNA through water (around 4.5 Å) [68–70]. While the direct contact peak is not affected significantly by crowding, the second peak shows a greater dependence on crowding. At the highest crowder fractions, the second peak is significantly reduced in both dodecamers (see Fig. 10b) and the ion density is reduced further at larger distances similar to the reduction in hydration upon crowding. The effect of crowding on the ion distributions also impacts the DNA neutralization as a function of distance (Fig. 10c). 76% of the DNA phosphate groups are neutralized as suggested by counterion condensation theory at around 9 Å for the dilute system, however, it takes up to 11–12 Å to reach 76% DNA neutralization under crowding conditions. It is interesting, that despite the impact of crowding on the second peak of the ion distribution, the counterion condensation is affected less for distances less than 6 Å. As this may seem counterintuitive, the reader is reminded that the RDF is normalized by the available volume and the overall ion density, at constant ion molality, whereas Fig. 10c simply describes the net neutralization of the DNA by the ions. The extended distance to reach 76% charge neutralization upon crowding may seem to challenge counterion condensation theory. However, the protein crowders, despite being net neutral, can provide additional charge neutralization by orienting basic lysines near the DNA surface as described above to compensate for the reduced neutralization by the sodium ions.

**Fig. 11:**
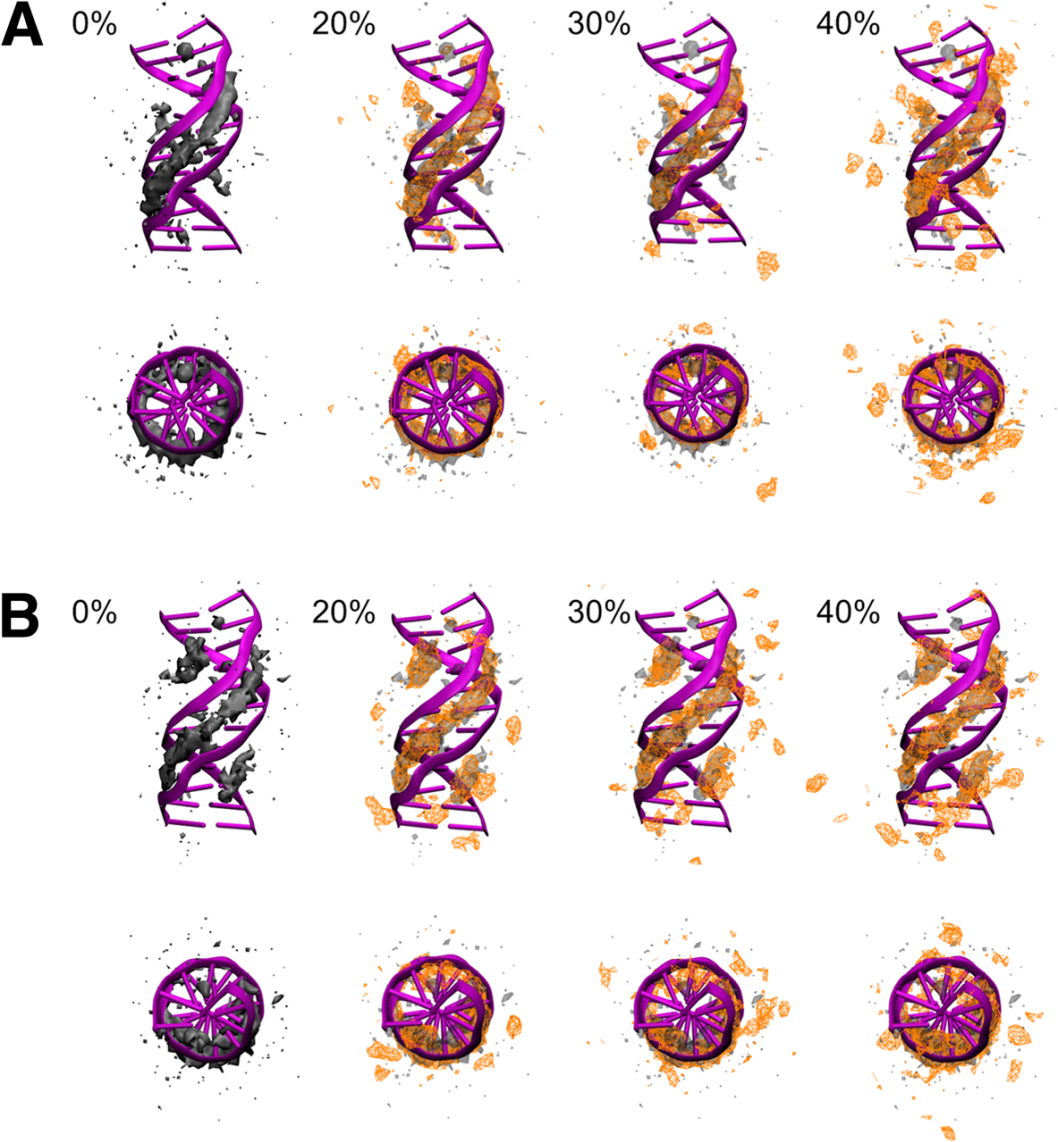
3D sodium ion densities around the Drew-Dickerson dodecamer (**a**) and the GC-rich dodecamer (**b**) at different protein concentrations. The ion density observed in 0% crowding is shown with a transparent representation for comparison with ion densities (orange) in crowded solutions. Density contours are shown at a level of 0.002 Å^− 3^. The top and bottom figures represent front and top views of DNA

Finally, the 3D distributions of sodium ions around the Drew-Dickerson and GC-rich dodecamers are compared in Fig. 11. The sodium ion networks in the major and minor grooves of DNA are largely preserved for both dodecamers with little changes upon crowding. However, additional densities become apparent further away from the DNA at different locations upon crowding. Additional ordering of ions could be a result of crowder proteins interacting with the DNA and coordinating ions near the DNA. A snapshot showing a crowder protein interacting with the DNA and orienting a sodium ion at the same time is shown in Additional file 1: Figure S19.

## Discussion

In this study, we investigated the effect of protein crowding on the conformational preferences of DNA duplexes. In a previous study, we examined one aspect of cellular crowding, namely a reduced dielectric response of the environment due to the less available water and its slowed dynamics. Using continuum models, we found an overall shift towards A-like conformations for DNA as a result of a reduced dielectric response of its environment [20]. Here, we included protein crowders and solvent explicitly to test whether the same conclusions would be found. In the earlier work, we compared environments with ε = 20, ε = 40, and ε = 80. Past work suggests that water under crowded conditions exhibits a reduced dielectric response of about 40 (with uncertainties) at a protein crowder volume fraction of 0.3 [18]. If one makes the further assumption that proteins have an interior dielectric of around 10, one can estimate an average effective dielectric for the entire medium surrounding the DNA at this crowder fraction as *ε*_*eff*_ = 0.3*10 + 0.7*40 = 31 with even lower values at 40% crowder fraction. However, although some of the base parameters moved slightly towards A-like values upon crowding, B-DNA was largely maintained with the explicit crowder environment in contrast to our previous findings. This suggests that a reduced dielectric response of crowded environments and interactions with crowder proteins have different effects on DNA conformational preferences with a net effect of not altering canonical B-DNA structures much.

We found that the crowder proteins mostly interact with DNA via its phosphate-sugar backbone as previously observed in non-specific binding of proteins to DNA [71]. These interactions arise from the electrostatic interactions between negatively charged phosphate oxygens and positively charged amino acid residues as well as the polar interactions between phosphate and/or sugar oxygens and side chains of polar amino acid residues. Previous studies have shown that DNA can undergo structural deformations from its B-form towards A-type helix as a result of forming complexes with specific DNA binding proteins [72–76], but we did not see such an effect here. It does appear, however, that for the system studied here, the presence of the protein crowders limits the conformational space of DNA to more canonical structures, mostly in B-form, both for the backbone torsions and the helical parameters. However, the narrowed conformational sampling appears to have little effect on the overall structural averages. Such a crowding effect on DNA structure may be understood in similar ways as protein native state stabilization due to the volume exclusion effect [1, 13, 77, 78], where the reduced space due to crowders limits the ability to widely sample conformational space. This would mean that protein crowding in vivo helps stabilize the biologically most relevant forms of DNA.

We also studied hydration patterns and ion densities around DNA in protein crowding. The first hydration shell around DNA is largely unaffected by crowding, while the water densities beyond the first solvation shell significantly reduced compared to the bulk water density under crowding effect. This result is very similar to the hydration shell around proteins upon crowding [18]. This further confirms that, protein crowding in cells generally does not alter the first hydration shell around biomolecules. However, sodium densities around DNA are affected already when interacting with DNA through water. Only the direct-contact first peak in the sodium-DNA RDF appears to be unaffected by crowding. Moreover, the charge neutralization by ions is altered upon crowding with the classical counter-ion condensation threshold reached at larger distances from the DNA than under dilute conditions. This suggests that proteins have to play an increasing role in neutralizing DNA under highly crowded conditions.

## Conclusion

The results obtained here shed light on the effect of protein crowding on DNA structure. We found that the crowder proteins mostly assist DNA to stay in canonical B-like conformations, limiting excursions to non-canonical conformations rather than a clear shift in the overall, average structure as suggested by a simple dielectric model of cellular environments. We hope that this hypothesis will motivate new experimental efforts to characterize DNA structure under crowded conditions. We expect that reduced conformational dynamics upon crowding would be observable via NMR spectroscopy. Another testable hypothesis is the altered ion distribution predicted by our simulations, which could be amenable to the ion-counting experiments recently carried out by the Herschlag group [79–82].

## Additional file

Additional file 1: Definition of backbone torsions and helicoidal parameters (**Figure S1**). Time series of helicoidal parameters (**Figure S2**). Potential of mean force (kcal/mol) as a function of backbone angles (**Figure S3**). Analysis of the protein G crowder conformational sampling (**Figure S4**). Average minimum distances between the crowder protein residues and DNA (**Figures S5-S6**). Potentials of mean force as a function of helicoidal parameters and protein contacts (**Figures S7-S18**). A snapshot for the crowder protein interacting with the DNA and sodium (**Figure S19**). Helicoidal parameters for the clusters (**Table S1 and S2**), bending angles for the dodecamers (**Table S3**), clustering analysis of protein G crowders (**Table S4**), and PMF error analysis (**Table S5**). (PDF 5597 kb)

## Abbreviations

ABNR: Adopted-bases Newton-Raphson
BSA: Bovine serum albumin
CHARMM: Chemistry at Harvard Molecular Mechanics
DNA: Deoxyribonucleic acid
MD: Molecular dynamics
MMTSB: Molecular Modeling Tools in Structural Biology
PEG: Polyethylene glycol
PMF: Potential of mean force
RDF: Radial distribution function
RMSD: Root mean square deviation
RMSF: Root mean square fluctuations
TIP3P: Three-site transferable intermolecular potential
VMD: Visual molecular dynamics
