# Supplementary material for: Role of protein interactions in stabilizing canonical DNA features in simulations of DNA in crowded environments
Source: BMC Biophys. 2018 Dec 7;11:8. doi: 10.1186/s13628-018-0048-y (PMC6286541; doi:10.1186/s13628-018-0048-y)
Supplement: Supplementary file 1 — Definition of backbone torsions and helicoidal parameters (Figure S1). Time series of helicoidal parameters (Figure S2). Potential of mean force (kcal/mol) as a function of backbone angles (Figure S3). Analysis of the protein G crowder conformational sampling (Figure S4). Average minimum distances between the crowder protein residues and DNA (Figures S5-S6). Potentials of mean force as a function of helicoidal parameters and protein contacts (Figures S7-S18). A snapshot for the crowder protein interacting with the DNA and sodium (Figure S19). Helicoidal parameters for the clusters (Table S1 and S2), bending angles for the dodecamers (Table S3), clustering analysis of protein G crowders (Table S4), and PMF error analysis (Table S5). (PDF 5597 kb) [file 13628_2018_48_MOESM1_ESM.pdf]

Supplementary Information

Role of Protein Interactions in Stabilizing Canonical DNA Features in Simulations of DNA in Crowded Environments

Asli Yildirim[1], Nathalie Brenner[2,3], Robert Sutherland[3], Michael Feig[3,]*

[1]Department of Chemistry, Michigan State University, East Lansing, Michigan 48824, USA

[2]Faculty of Mathematics and Natural Sciences, University of Düsseldorf, 40225 Düsseldorf, Germany

[3]Department of Biochemistry & Molecular Biology, Michigan State University, East Lansing, Michigan 48824, USA

Corresponding Author Contact Information

[*]603 Wilson Road
Room BCH 218
East Lansing, MI
48824
USA
Phone: 517-432-7439
E-mail: feig@msu.edu

Table S1. Parameters and RMSD values from the canonical B-form structure for the individual clusters of the Drew-Dickerson dodecamer.

	**DD1**	**DD2**	**DD3**	**DD4**
Slide (Å)	0.07 (*0.00*)	0.34 (*0.00*)	0.19 (*0.00*)	0.38 (*0.00*)
Twist (deg)	33.67 (*0.01*)	34.63 (*0.01*)	33.12 (*0.01*)	32.73 (*0.02*)
X-displacement (Å)	-1.09 (*0.00*)	-0.30 (*0.01*)	-0.94 (*0.01*)	-0.42 (*0.01*)
Helical rise (Å)	3.21 (*0.00*)	3.30 (*0.00*)	3.24 (*0.00*)	3.29 (*0.00*)
Inclination (deg)	12.89 (*0.03*)	8.95 (*0.03*)	13.14 (*0.04*)	11.07 (*0.05*)
z_p (Å)	-0.06 (*0.00*)	-0.32 (*0.00*)	-0.14 (*0.00*)	-0.29 (*0.00*)
Minor groove (Å)	13.63 (*0.01*)	12.77 (*0.01*)	13.86 (*0.01*)	13.69 (*0.01*)
Major groove (Å)	16.10 (*0.01*)	16.52 (*0.01*)	16.27 (*0.01*)	16.97 (*0.01*)
RMSD (Å)	1.82 (*0.00*)	1.37 (*0.00*)	2.00 (*0.00*)	1.70 (*0.00*)

All values are averaged over all base-pairs excluding the first and last two terminal base-pairs with standard errors given in the parentheses.

Table S2. Parameters and RMSD values from the canonical B-form structure for the individual clusters of the GC-rich dodecamer.

	**GC1**	**GC2**	**GC3**	**GC4**
Slide (Å)	0.12 (*0.00*)	-0.30 (*0.00*)	0.69 (*0.01*)	0.44 (*0.01*)
Twist (deg)	34.36 (*0.01*)	33.13 (*0.01)*	34.63 (*0.03*)	33.02 (*0.03*)
X-displacement (Å)	-0.67 (*0.01*)	-1.69 (*0.01*)	0.53 (*0.01*)	-0.20 (*0.01*)
Helical rise (Å)	3.30 (*0.00*)	3.20 (*0.00)*	3.37 (*0.00*)	3.33 (*0.00*)
Inclination (deg)	8.80 (*0.03*)	12.12 (*0.03*)	5.90 (*0.08*)	9.42 (*0.07*)
z_p (Å)	0.05 (*0.00*)	0.34 (*0.00*)	-0.21 (*0.01*)	-0.08 (*0.01*)
Minor groove (Å)	14.05 (*0.01*)	14.55 (*0.01*)	14.00 (*0.02*)	14.78 (*0.01*)
Major groove (Å)	16.34 (*0.01*)	16.13 (*0.01*)	16.47 (*0.01*)	16.82 (*0.02*)
RMSD (Å)	1.62 (*0.00*)	2.28 (*0.00*)	1.68 (*0.01*)	2.05 (*0.01*)
	**GC5**	**GC6**	**GC7**	**GC8**
Slide (Å)	0.44 (*0.01*)	-0.06 (*0.01*)	-0.11 (*0.02*)	0.04 (*0.00*)
Twist (deg)	30.98 (*0.06*)	31.97 (*0.03*)	30.90 (*0.41*)	32.72 (*0.02*)
X-displacement (Å)	-0.55 (*0.02*)	-1.41 (*0.01*)	-0.94 (*0.03*)	-1.19 (*0.01*)
Helical rise (Å)	3.36 (*0.01*)	3.24 (*0.00*)	3.07 (*0.02*)	3.25 (*0.00*)
Inclination (deg)	12.55 (*0.14*)	13.05 (*0.06*)	12.00 (*0.30*)	12.99 (*0.04*)
z_p (Å)	0.03 (*0.01*)	0.19 (*0.01*)	-0.20 (*0.03*)	0.17 (*0.01*)
Minor groove (Å)	15.68 (*0.02*)	15.11 (*0.01*)	14.94 (*0.03*)	15.09 (*0.01*)
Major groove (Å)	16.82 (*0.02*)	16.69 (*0.02*)	15.47 (*0.05*)	16.25 (*0.01*)
RMSD (Å)	2.58 (*0.01*)	2.47 (*0.01*)	3.70 (*0.02*)	2.25 (*0.00*)

All values are averaged over all base-pairs excluding the first and last two terminal base-pairs with standard errors given in the parentheses.

Table S3. Bending angles for both DNA dodecamers (degrees).

		Canonical		Simulations in crowded environment			
	X-ray	A-DNA	B-DNA	0%	20%	30%	40%
Drew-Dickerson	170.11	122.72 (*3.57*)	163.05 (*5.94*)	155.24 (*0.47*)	155.55 (*0.65*)	155.50 (*0.76*)	152.80 (*0.87*)
GC-rich	100.77	122.72 (*3.57*)	163.05 (*5.94*)	156.85 (*1.09*)	153.00 (*1.12*)	153.03 (*0.91*)	152.18 (*1.04*)

Bending angle is defined as the angle between the center of masses of three sections of base-pairs: Section 1: 3 – 5, Section 2: 6 – 7, and Section 3: 8 – 10. Standard errors given in the parentheses. Statistical errors of the averages over the simulations are estimated from block averaging by comparing results for 100 ns segments from the simulations. Canonical values are averaged over the A-form structures 3V9D, 3QK4, 2B1B, 1ZEX, 1ZEY, 1ZF1, 1ZF8, 1ZF9, 1ZFA and the B-form structures 2M2C, 4AGZ, 4H0, 4AH1, 3U05, 3U08, 1VTJ,3U2N, 3OIE, 3BSE. For X-ray structure values; 1BNA and 399D are used for Drew-Dickerson and GC-rich dodecamers, respectively.

Table S4. Conformational clustering of protein G crowder molecules

		Cluster	Population	Cluster center		Avg. minimum DNA distance [Å]
				Cα RMSD [Å]	Radius of gyration [Å]	
Drew-Dickerson	20%	1	76.9 %	0.57	10.53	7.9
		2	21.6 %	0.73	10.67	6.9
		3	1.2 %	1.18	10.61	4.2
		4	0.3%	2.32	10.49	4.3
	30%	1	68.8 %	0.57	10.57	7.2
		2	28.1 %	0.76	10.56	7.1
		3	3.1 %	1.45	10.71	5.3
	40%	1	63.1 %	0.63	10.59	6.4
		2	36.9 %	0.91	10.76	6.5
GC-rich	20%	1	95.6 %	0.64	10.59	8.0
		2	4.4 %	2.07	10.62	3.8
	30%	1	100 %	0.65	10.63	6.0
	40%	1	94.2 %	0.59	10.59	6.3
		2	5.8 %	1.14	10.70	3.7

Clustering analysis of snapshots extracted at 10 ns intervals via the kclust program of the MMTSB Tool Set based on Cα atoms using a clustering radius of 2 Å. Cα RMSD is with respect to experimental structure (3GB1). Minimum DNA distance is calculated between heavy atoms of protein and DNA.

Table S5. PMF error analysis

	Figure	Drew-Dickerson			GC-rich		
		20%	30%	40%	20%	30%	40%
slide	6	0.21<br>[-0.8; 0.5]	0.30<br>[-0.5; 0.8]	0.34<br>[-0.8; 0.7]	0.39<br>[-0.6; 1.3]	0.41<br>[-1.3; 1.0]	0.41<br>[-1.3; 1.0]
x-displacement	7	0.20<br>[-0.6; 0.3]	0.25<br>[-0.2; 0.7]	0.32<br>[-0.6; 0.5]	0.41<br>[-0.6; 1.2]	0.40<br>[-1.1; 1.0]	0.39<br>[-0.7; 1.3]
helical rise	8	0.20<br>[-0.4; 0.4]	0.22<br>[-0.5; 0.6]	0.32<br>[-0.7; 0.6]	0.35<br>[-0.7; 1.0]	0.30<br>[-0.9; 0.9]	0.40<br>[-0.7; 1.5]
z_p	9	0.22<br>[-0.9; 0.6]	0.23<br>[-0.5; 0.6]	0.31<br>[-0.8; 0.6]	0.42<br>[-1.1; 1.2]	0.38<br>[-1.0; 1.2]	0.41<br>[-0.8; 1.4]
twist	S7	0.22<br>[-0.4; 0.8]	0.22<br>[-0.7; 0.6]	0.32<br>[-0.7; 0.7]	0.36<br>[-0.7; 1.0]	0.26<br>[-0.7; 0.8]	0.39<br>[-0.3; 1.3]
inclination	S8	0.23<br>[-0.8; 0.6]	0.29<br>[-0.8; 0.9]	0.34<br>[-1.1; 0.8]	0.38<br>[-0.8; 1.2]	0.34<br>[-1.2; 1.1]	0.37<br>[-0.6; 1.4]
minor groove	S9	0.41<br>[-1.3; 1.3]	0.35<br>[-0.6; 1.2]	0.42<br>[-1.0; 1.1]	0.43<br>[-1.2; 1.6]	0.43<br>[-1.5; 1.4]	0.45<br>[-1.2; 1.4]
major groove	S10	0.27<br>[-0.9; 0.9]	0.38<br>[-1.3; 1.3]	0.41<br>[-1.3; 1.1]	0.46<br>[-1.3; 2.0]	0.41<br>[-1.1; 1.5]	0.50<br>[-1.1; 1.6]
α	S11	0.22<br>[-0.7; 0.8]	0.23<br>[-0.3; 0.8]	0.31<br>[-0.7; 0.7]	0.35<br>[-0.8; 1.2]	0.25<br>[-0.6; 0.8]	0.55<br>[-0.5; 2.0]
β	S12	0.22<br>[-0.5; 0.9]	0.22<br>[-0.5; 0.7]	0.34<br>[-0.9; 0.7]	0.42<br>[-0.8; 1.7]	0.31<br>[-0.9; 1.0]	0.49<br>[-0.8; 2.2]
γ	S13	0.20<br>[-0.2; 0.5]	0.19<br>[-0.1; 0.5]	0.33<br>[-0.9; 0.6]	0.36<br>[-0.7; 1.3]	0.27<br>[-0.8; 0.7]	0.53<br>[-0.3; 2.1]
δ	S14	0.22<br>[-0.3; 0.6]	0.23<br>[-0.4; 0.8]	0.33<br>[-0.8; 0.7]	0.37<br>[-0.7; 1.2]	0.30<br>[-0.8; 0.7]	0.38<br>[-0.5; 1.5]
ε	S15	0.21<br>[-0.6; 0.6]	0.26<br>[-0.6; 0.8]	0.31<br>[-0.7; 0.6]	0.33<br>[-0.8; 1.4]	0.29<br>[-1.1; 0.6]	0.29<br>[-0.5; 1.2]
ζ	S16	0.25<br>[-1.0; 0.8]	0.30<br>[-0.6; 0.8]	0.29<br>[-0.9; 0.7]	0.39<br>[-1.0; 1.3]	0.30<br>[-0.9; 0.9]	0.35<br>[-1.0; 1.2]
χ	S17	0.25<br>[-0.9; 1.0]	0.26<br>[-0.2; 0.8]	0.32<br>[-0.7; 0.7]	0.40<br>[-0.6; 1.6]	0.37<br>[-1.1; 0.9]	0.39<br>[-1.0; 1.2]
pucker	S18	0.26<br>[-0.6; 0.9]	0.29<br>[-0.6; 1.1]	0.33<br>[-1.0; 0.6]	0.38<br>[-1.0; 1.2]	0.31<br>[-0.9; 0.9]	0.40<br>[-0.9; 1.5]
δ/χ	3	0.16<br>[-0.6; 0.1]	0.14<br>[-0.2; 0.4]	0.16<br>[-0.0; 0.4]	0.29<br>[-0.2; 1.1]	0.24<br>[-0.6; 0.3]	0.19<br>[-0.4; 0.5]
ε/ζ	S3	0.19<br>[-0.9; 0.5]	0.24<br>[-0.4; 1.4]	0.22<br>[-0.3; 0.7]	0.20<br>[-0.7; 0.7]	0.24<br>[-0.7; 0.2]	0.22<br>[-0.7; 1.0]

Errors in kcal/mol estimated from comparing PMFs generated from first (300-650 ns) and second (650-1000 ns) halves of production trajectories. The first value corresponds to the root mean square deviation across all PMF grid points where PMF energies are less than 4 kcal/mol. Values in square brackets are minimum and maximum deviations.

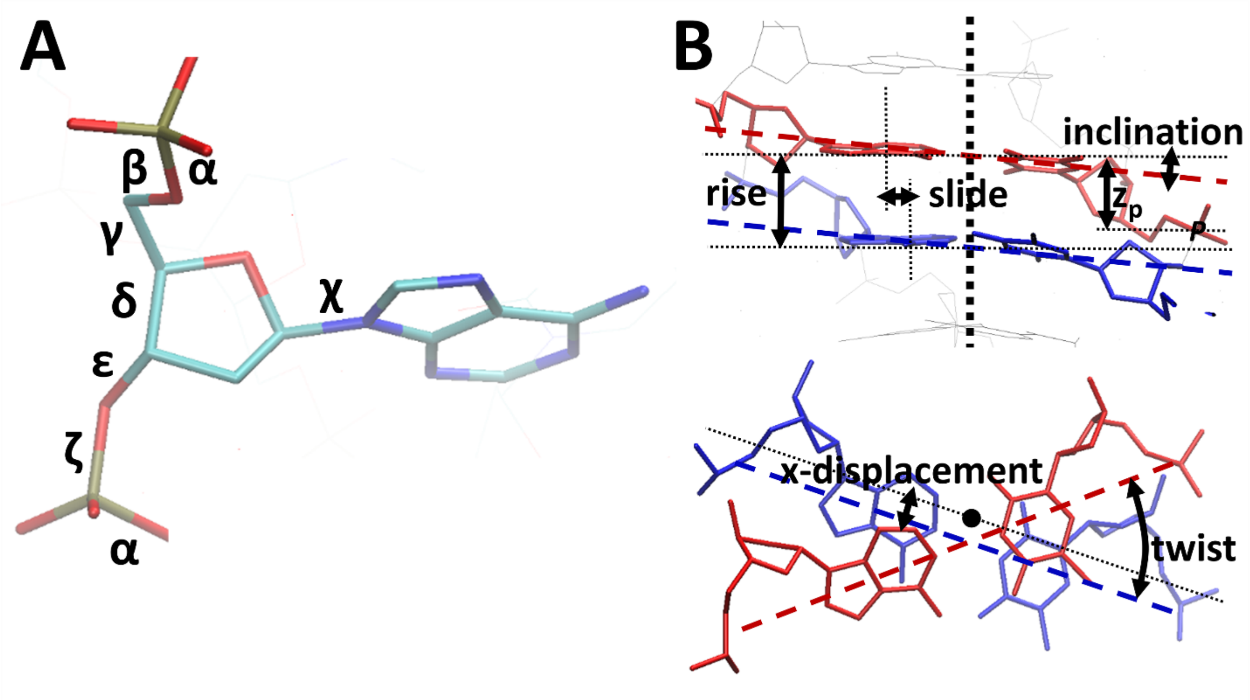

Figure S1. Definitions of DNA backbone torsion angles (A) and helical base pair parameters discussed in this study (B).

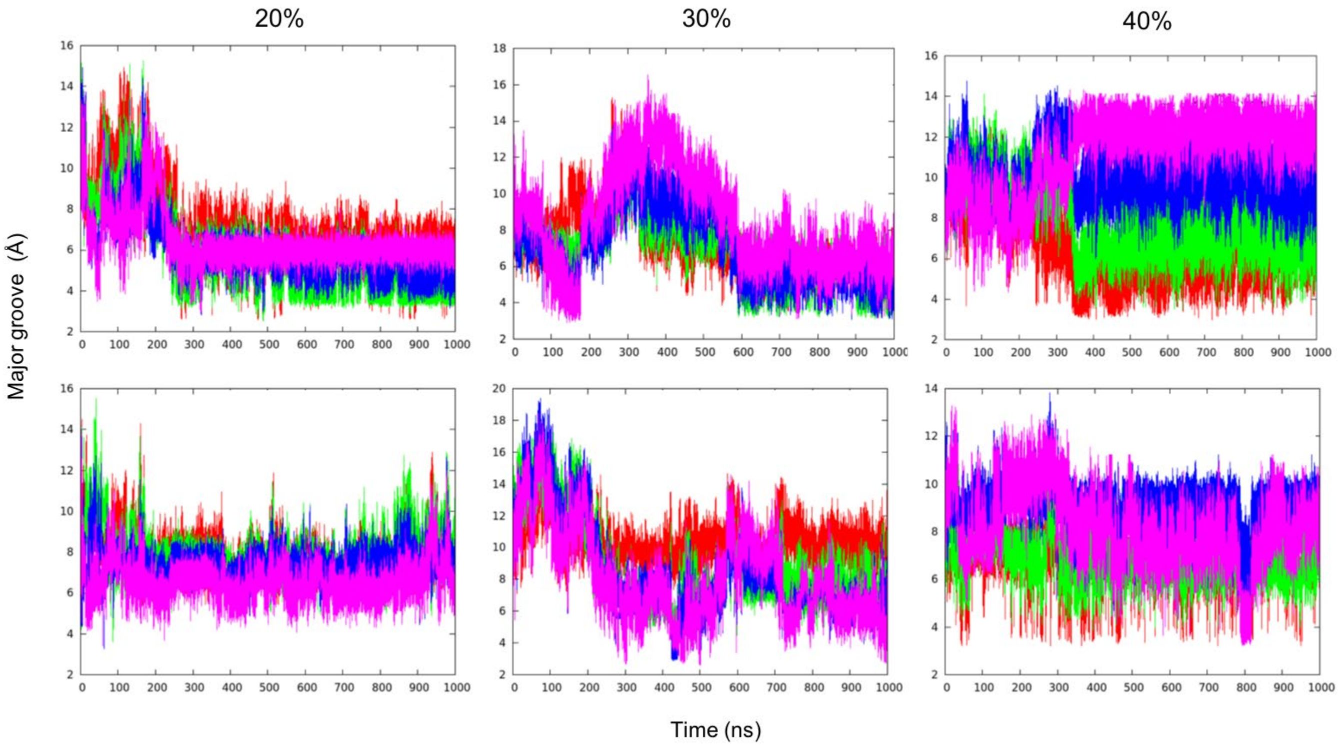

Figure S2. Time series of major groove widths for inner 5th (red), 6th (green), 7th (blue) and 8th (pink) basepairs for the Drew-Dickerson (top) and GC-rich dodecamers in crowded simulations.

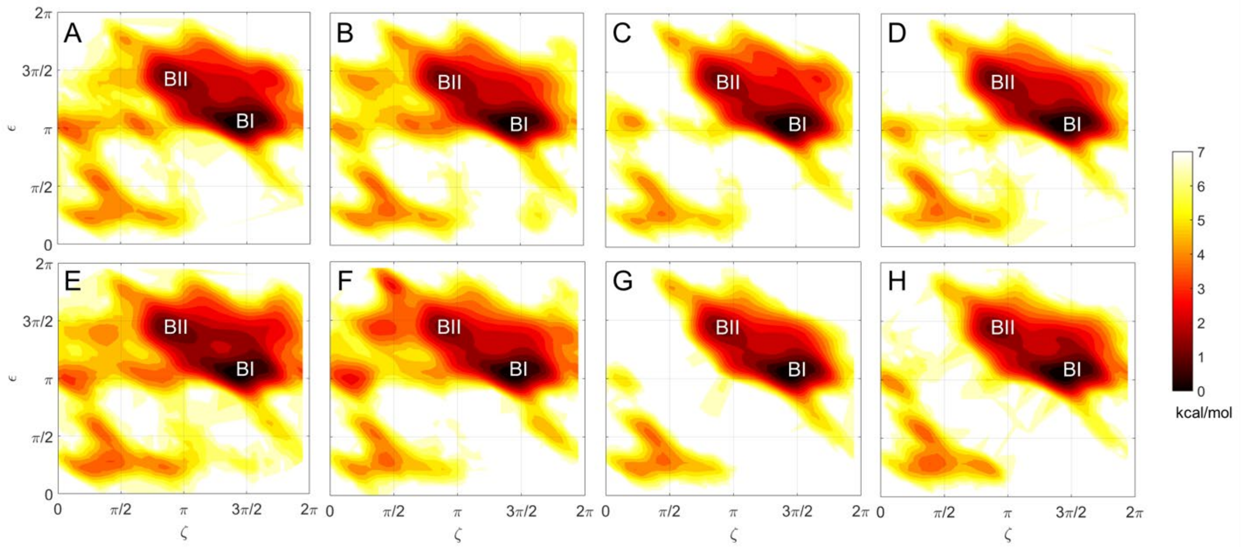

Figure S3. Potential of mean force (kcal/mol) as a function of ε and ξ backbone angles for the Drew-Dickerson dodecamer at 0 % (A), 20 % (B), 30 % (C) and 40 % (D) protein concentrations, and for the GC-rich dodecamer at 0 % (E), 20 % (F), 30 % (G) and 40 % (H) protein concentrations. See Table S5 for uncertainties.

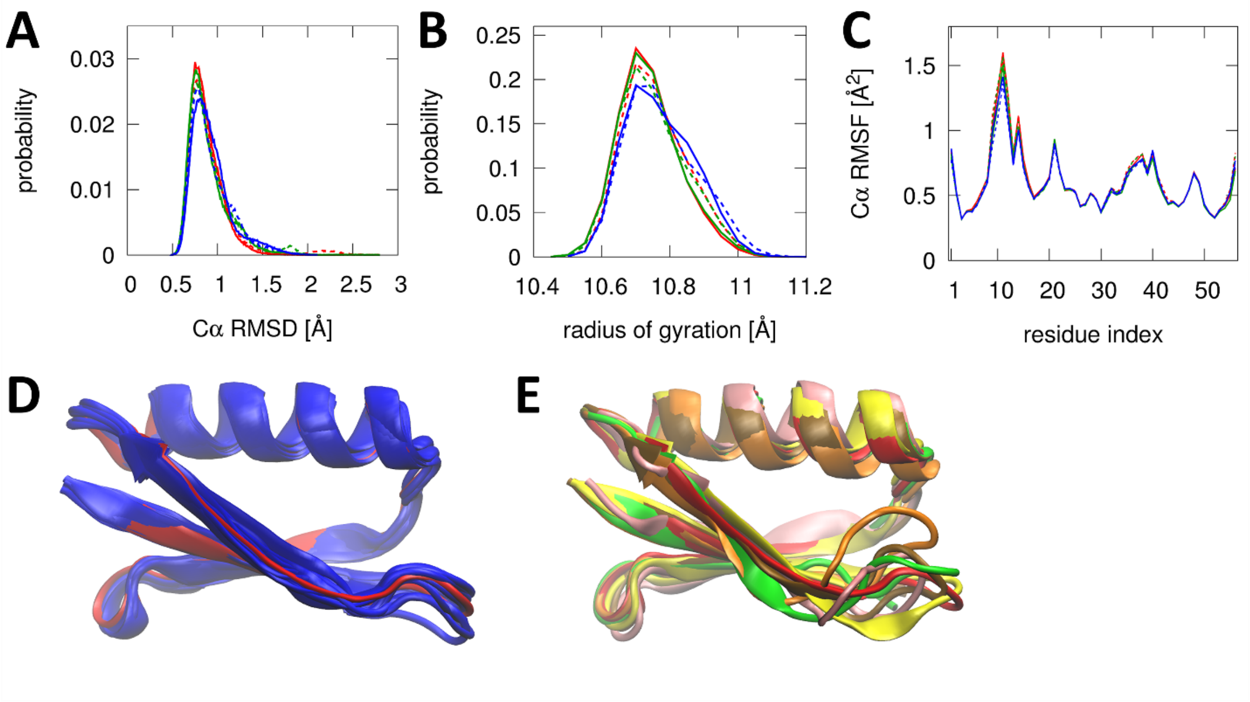

Figure S4. Conformational analysis of protein G crowders for 20% (red), 30% (green), and 40% (blue) crowder concentrations around the Drew-Dickerson dodecamer (solid lines) and the GC-rich DNA (dashed lines) (A-C). Distribution of Cα RMSD with respect to experimental structure (3GB1) (A); distribution of radius of gyration (B); averaged root mean square fluctuations for Cα atoms as a function of protein G residue. Representative structures from cluster analysis for highly populated clusters (blue, D) and minor substates (colors, E), compared with experimental structure (red).

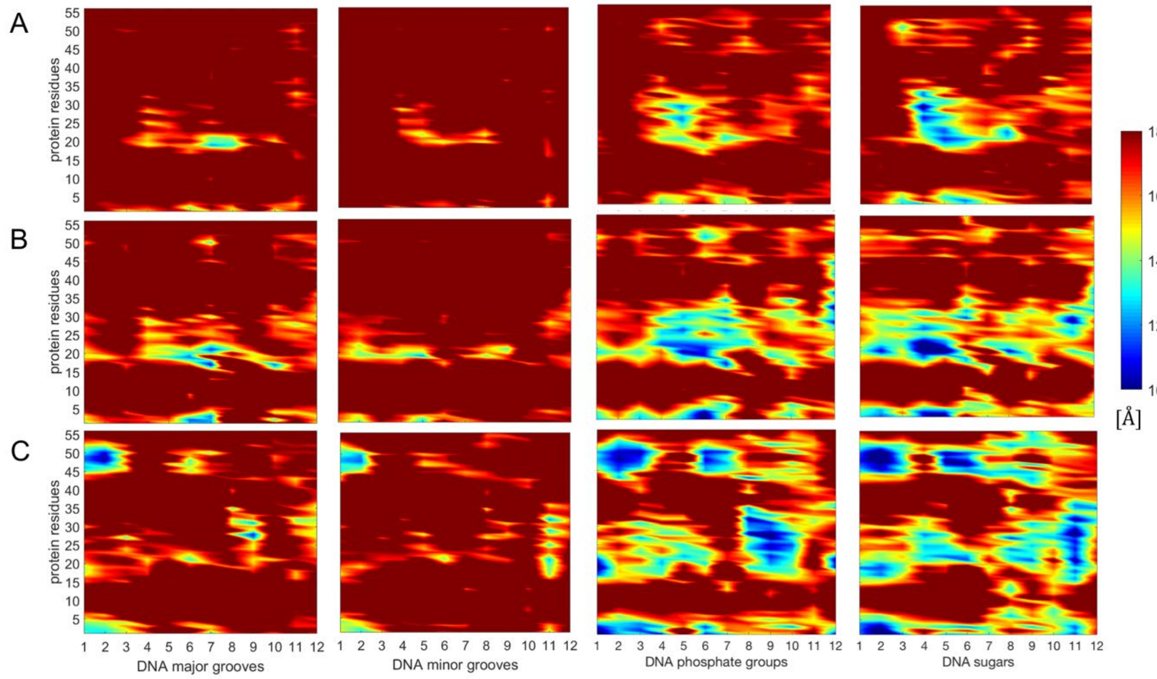

Figure S5. Average minimum distances between the crowder protein residues and the major groove, minor groove, sugar and phosphate backbone for the individual base-pairs of Drew-Dickerson dodecamer at 20% (A), 30% (B) and 40% (C) protein concentrations. Distances were calculated from the heavy atoms only.

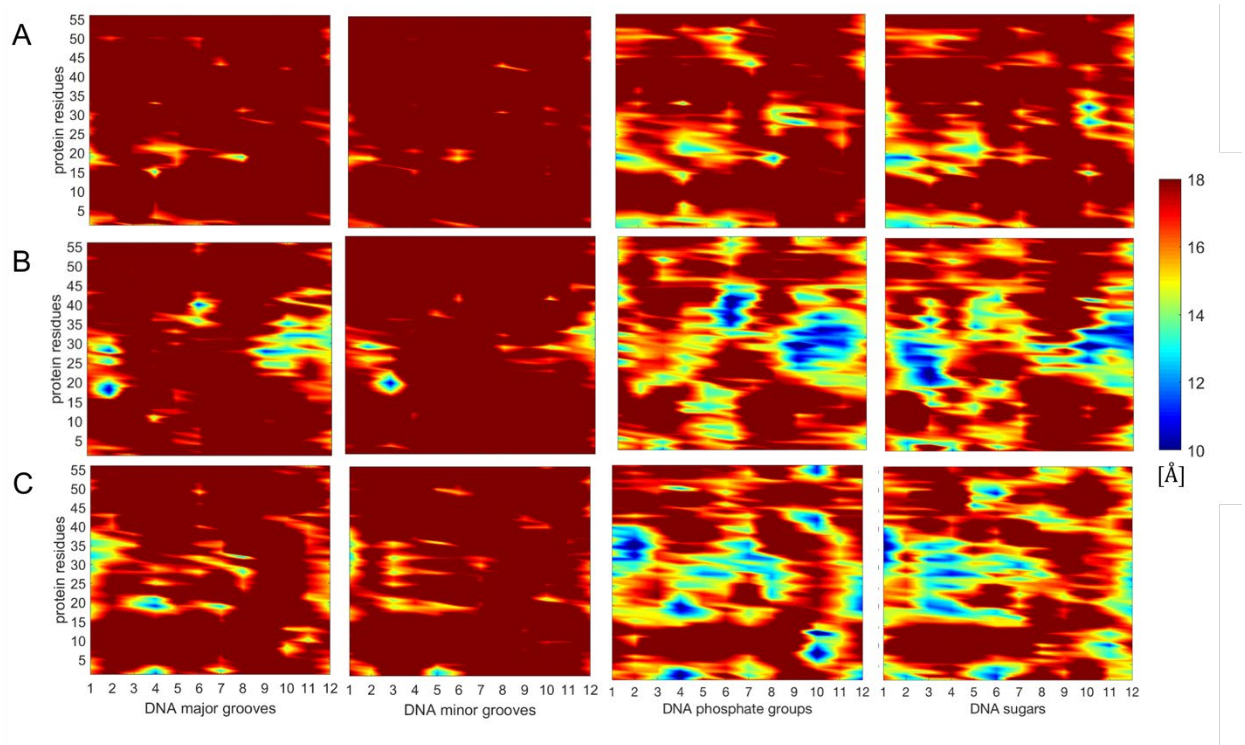

Figure S6. Average minimum distances between the crowder protein residues and the major grooves, minor grooves, sugar and phosphate backbone for the individual base-pairs of GC-rich dodecamer at 20% (A), 30% (B) and 40% (C) protein concentrations. Distances were calculated from the heavy atoms only.

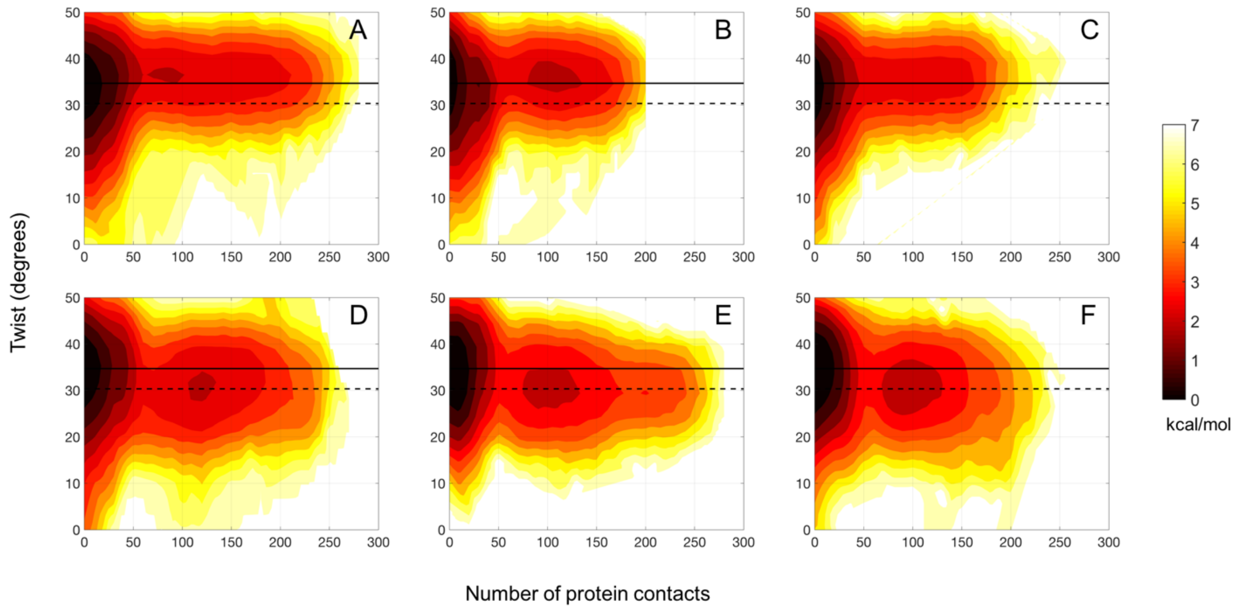

Figure S7. Potential of mean force (kcal/mol) as a function of twist angle and number of protein contacts for the Drew-Dickerson dodecamer at 20 % (A), 30 % (B), 40 % (C) protein concentrations, and for the GC-rich dodecamer at 20 % (D), 30 % (E), 40 % (F) protein concentrations. A contact is defined when the minimum distance between the heavy atoms of crowder proteins and DNA phosphate groups is less than 5 Å. Solid and dashed lines indicate the canonical B- and A-form values, respectively. See Table S5 for uncertainties.

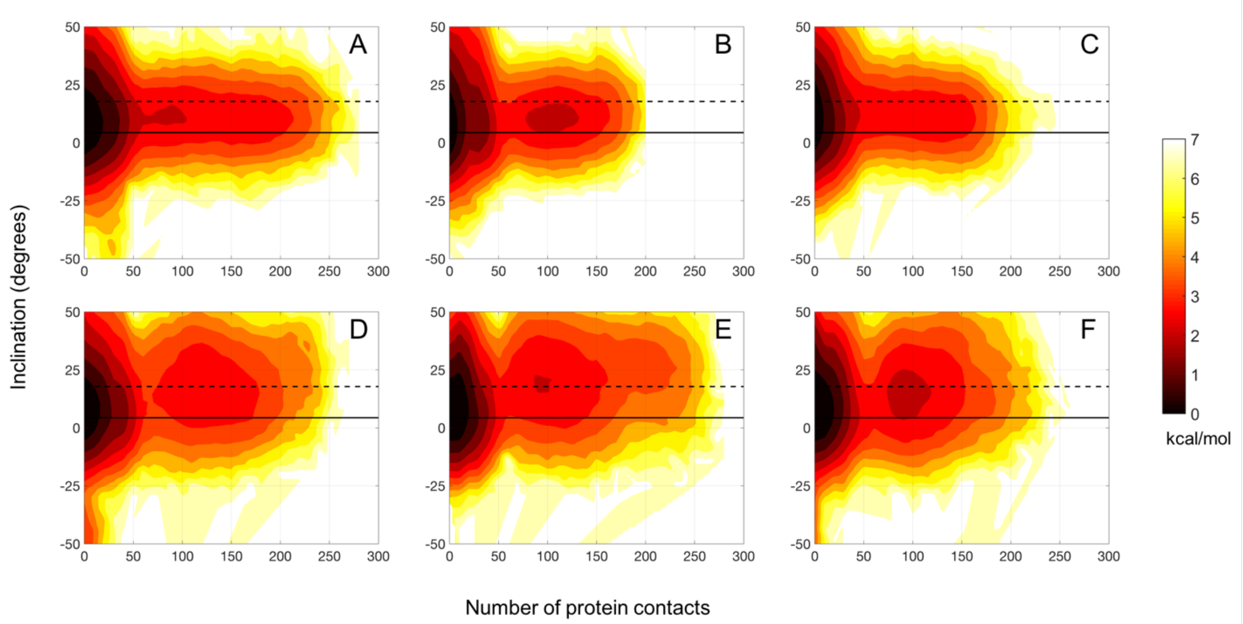

Figure S8. Potential of mean force (kcal/mol) as a function of inclination angle and number of protein contacts for the Drew-Dickerson dodecamer at 20 % (A), 30 % (B), 40 % (C) protein concentrations, and for the GC-rich dodecamer at 20 % (D), 30 % (E), 40 % (F) protein concentrations. A contact is defined when the minimum distance between the heavy atoms of crowder proteins and DNA phosphate groups is less than 5 Å. Solid and dashed lines indicate the canonical B- and A-form values, respectively. See Table S5 for uncertainties.

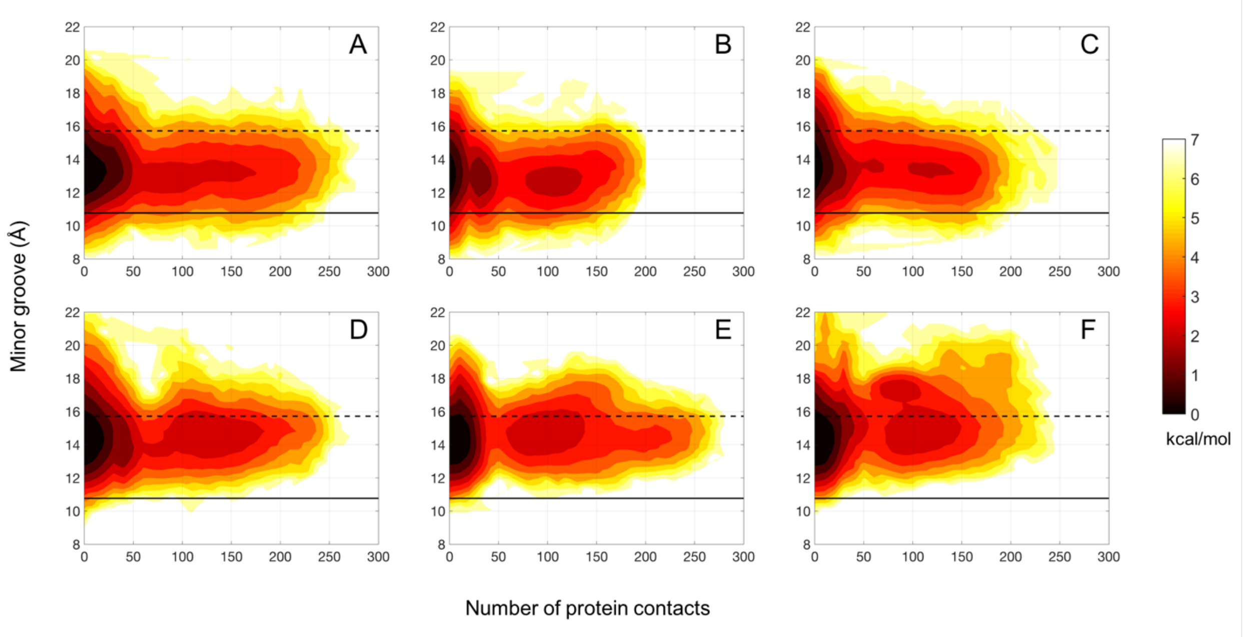

Figure S9. Potential of mean force (kcal/mol) as a function of minor groove width and number of protein contacts for the Drew-Dickerson dodecamer at 20 % (A), 30 % (B), 40 % (C) protein concentrations, and for the GC-rich dodecamer at 20 % (D), 30 % (E), 40 % (F) protein concentrations. A contact is defined when the minimum distance between the heavy atoms of crowder proteins and DNA phosphate groups is less than 5 Å. Solid and dashed lines indicate the canonical B- and A-form values, respectively. See Table S5 for uncertainties.

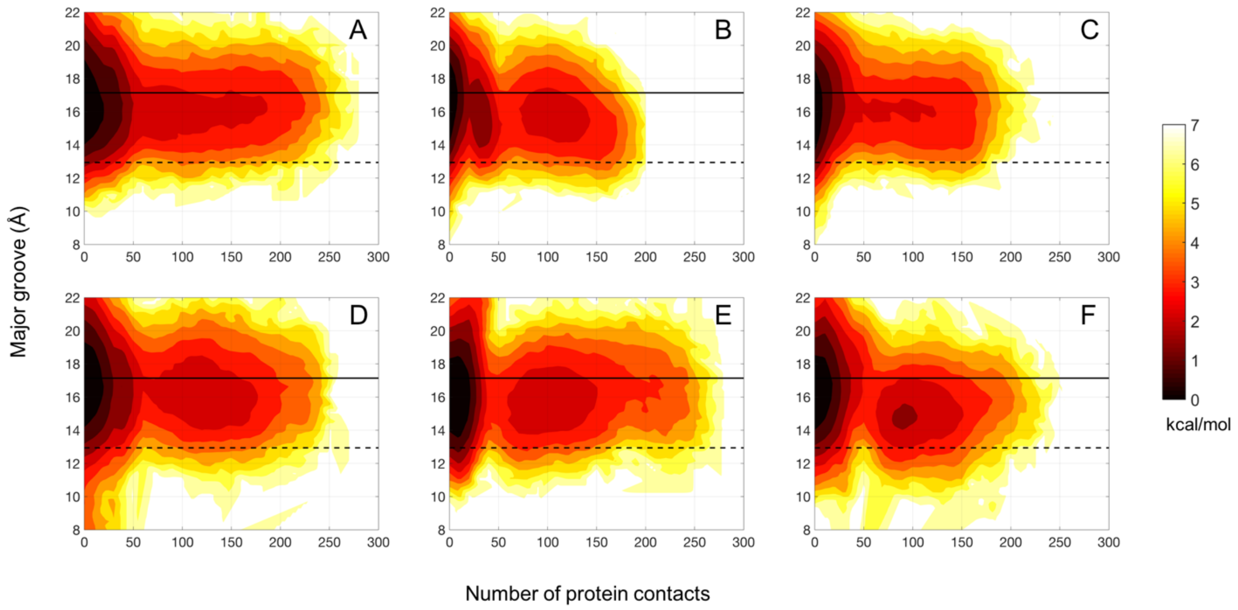

Figure S10. Potential of mean force (kcal/mol) as a function of major groove width and number of protein contacts for the Drew-Dickerson dodecamer at 20 % (A), 30 % (B), 40 % (C) protein concentrations, and for the GC-rich dodecamer at 20 % (D), 30 % (E), 40 % (F) protein concentrations. A contact is defined when the minimum distance between the heavy atoms of crowder proteins and DNA phosphate groups is less than 5 Å. Solid and dashed lines indicate the canonical B- and A-form values, respectively. See Table S5 for uncertainties.

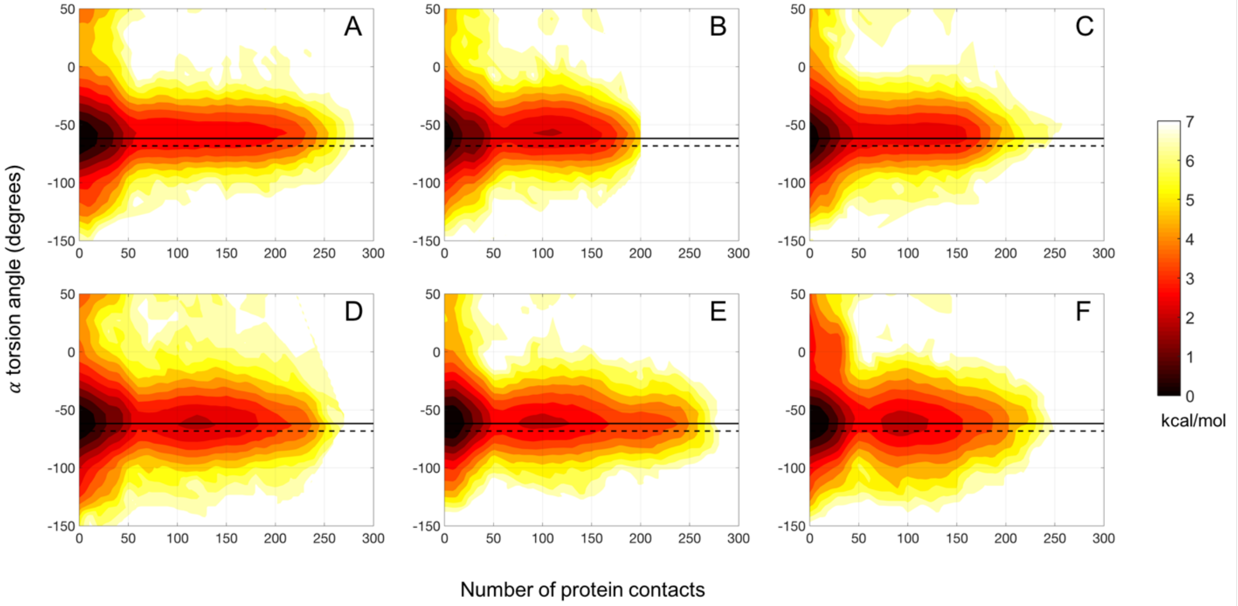

Figure S11. Potential of mean force (kcal/mol) as a function of α backbone torsion angle and number of protein contacts for the Drew-Dickerson dodecamer at 20 % (A), 30 % (B), 40 % (C) protein concentrations, and for the GC-rich dodecamer at 20 % (D), 30 % (E), 40 % (F) protein concentrations. A contact is defined when the minimum distance between the heavy atoms of crowder proteins and DNA phosphate groups is less than 5 Å. Solid and dashed lines indicate the canonical B- and A-form values, respectively. See Table S5 for uncertainties.

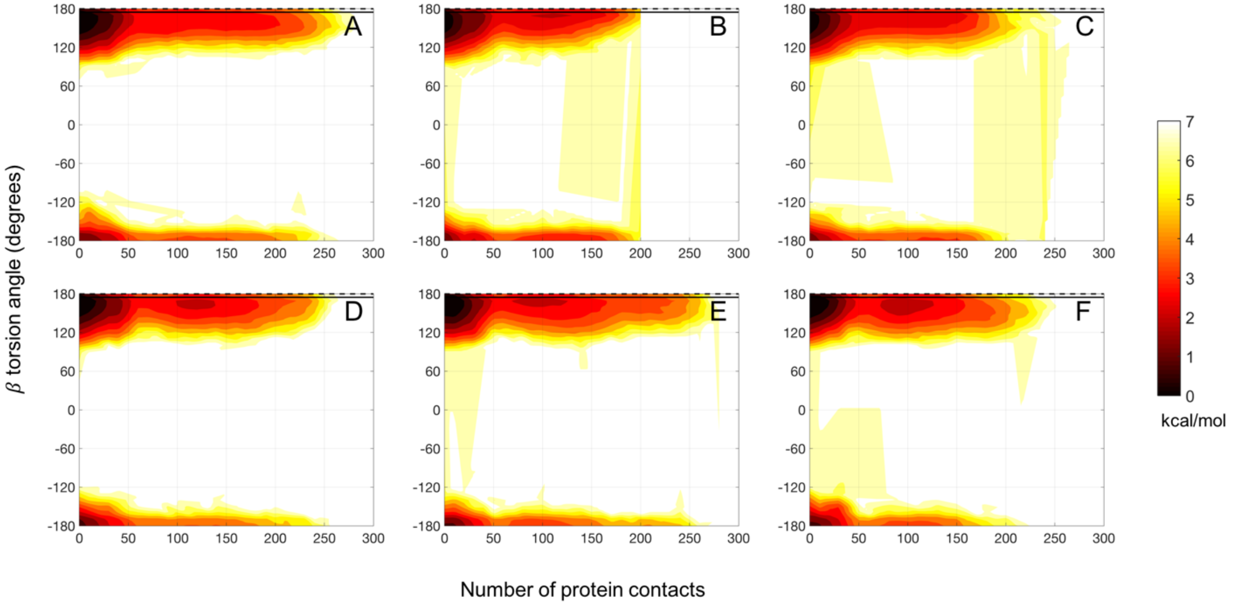

Figure S12. Potential of mean force (kcal/mol) as a function of β backbone torsion angle and number of protein contacts for the Drew-Dickerson dodecamer at 20 % (A), 30 % (B), 40 % (C) protein concentrations, and for the GC-rich dodecamer at 20 % (D), 30 % (E), 40 % (F) protein concentrations. A contact is defined when the minimum distance between the heavy atoms of crowder proteins and DNA phosphate groups is less than 5 Å. Solid and dashed lines indicate the canonical B- and A-form values, respectively. See Table S5 for uncertainties.

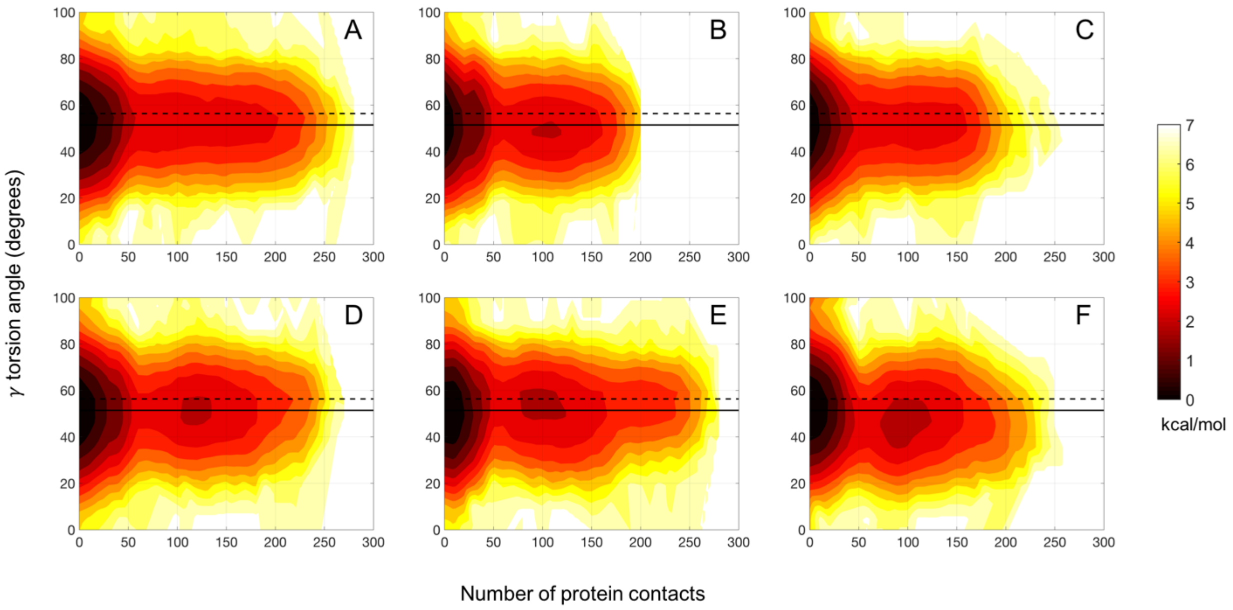

Figure S13. Potential of mean force (kcal/mol) as a function of γ backbone torsion angle and number of protein contacts for the Drew-Dickerson dodecamer at 20 % (A), 30 % (B), 40 % (C) protein concentrations, and for the GC-rich dodecamer at 20 % (D), 30 % (E), 40 % (F) protein concentrations. A contact is defined when the minimum distance between the heavy atoms of crowder proteins and DNA phosphate groups is less than 5 Å. Solid and dashed lines indicate the canonical B- and A-form values, respectively. See Table S5 for uncertainties.

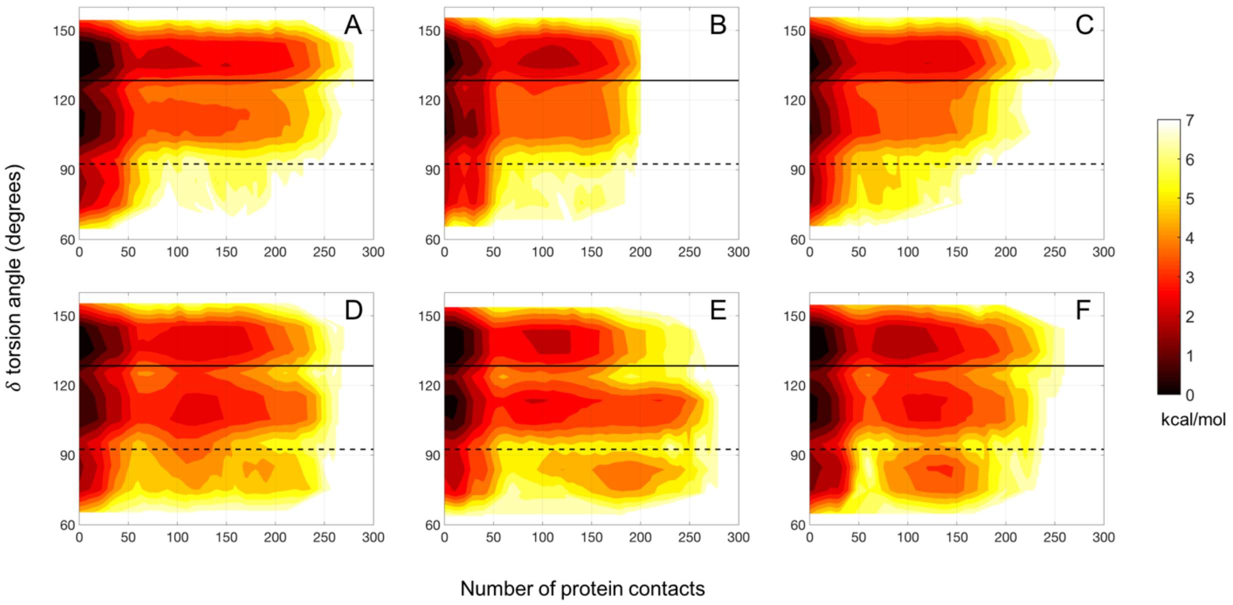

Figure S14. Potential of mean force (kcal/mol) as a function of δ backbone torsion angle and number of protein contacts for the Drew-Dickerson dodecamer at 20 % (A), 30 % (B), 40 % (C) protein concentrations, and for the GC-rich dodecamer at 20 % (D), 30 % (E), 40 % (F) protein concentrations. A contact is defined when the minimum distance between the heavy atoms of crowder proteins and DNA phosphate groups is less than 5 Å. Solid and dashed lines indicate the canonical B- and A-form values, respectively. See Table S5 for uncertainties.

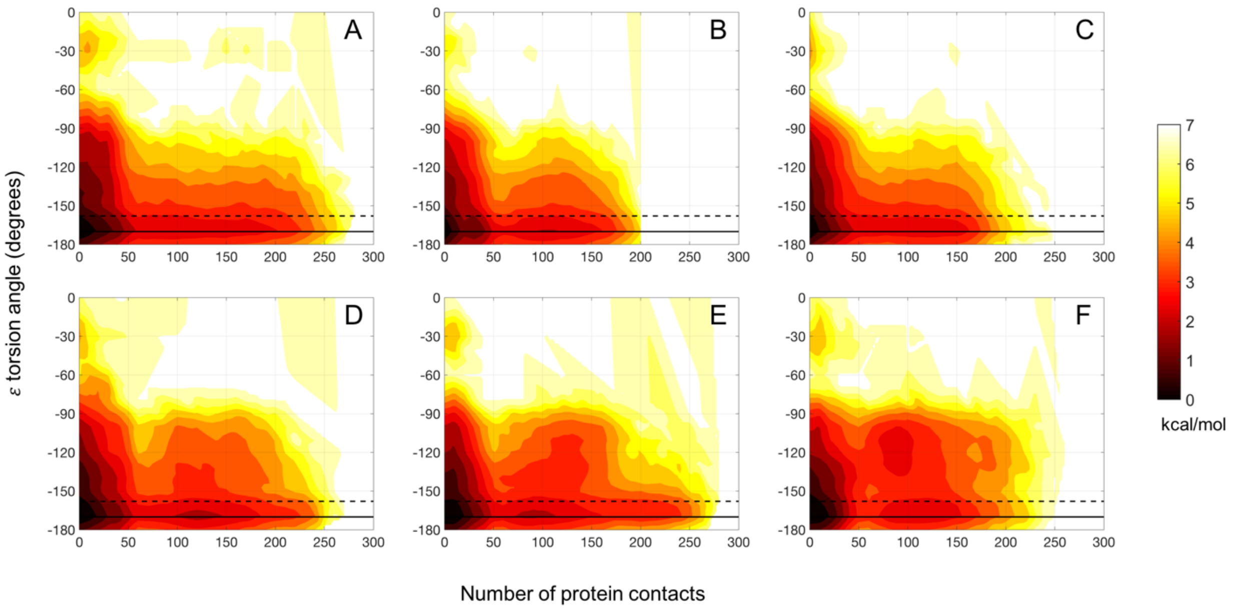

Figure S15. Potential of mean force (kcal/mol) as a function of ε backbone torsion angle and number of protein contacts for the Drew-Dickerson dodecamer at 20 % (A), 30 % (B), 40 % (C) protein concentrations, and for the GC-rich dodecamer at 20 % (D), 30 % (E), 40 % (F) protein concentrations. A contact is defined when the minimum distance between the heavy atoms of crowder proteins and DNA phosphate groups is less than 5 Å. Solid and dashed lines indicate the canonical B- and A-form values, respectively. See Table S5 for uncertainties.

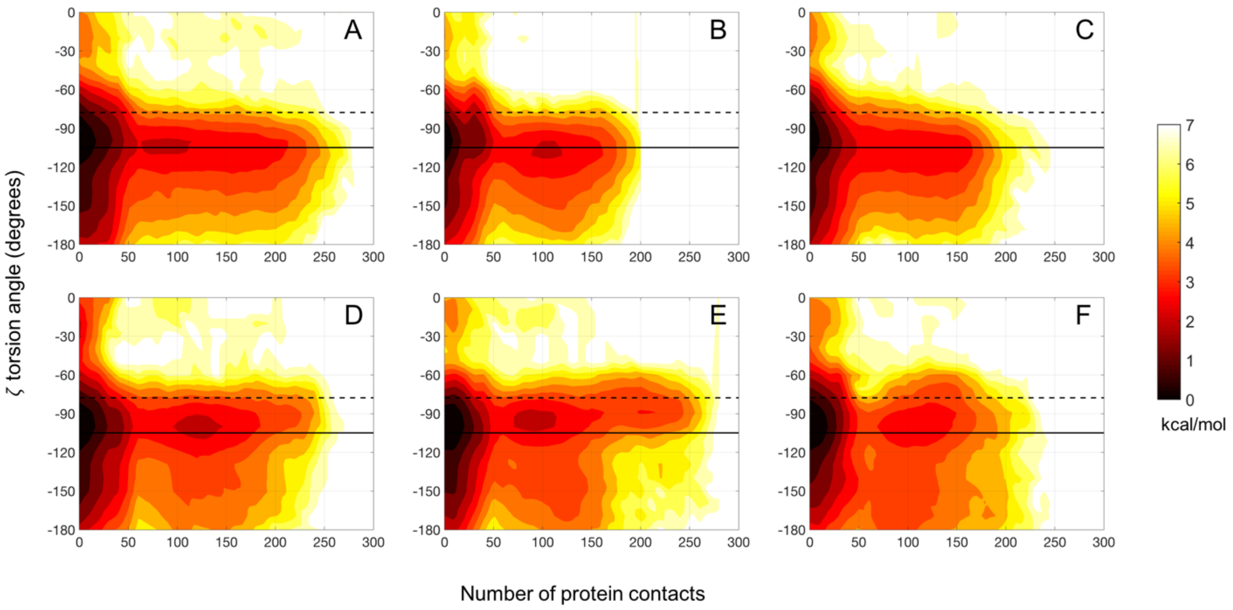

Figure S16. Potential of mean force (kcal/mol) as a function of ζ backbone torsion angle and number of protein contacts for the Drew-Dickerson dodecamer at 20 % (A), 30 % (B), 40 % (C) protein concentrations, and for the GC-rich dodecamer at 20 % (D), 30 % (E), 40 % (F) protein concentrations. A contact is defined when the minimum distance between the heavy atoms of crowder proteins and DNA phosphate groups is less than 5 Å. Solid and dashed lines indicate the canonical B- and A-form values, respectively. See Table S5 for uncertainties.

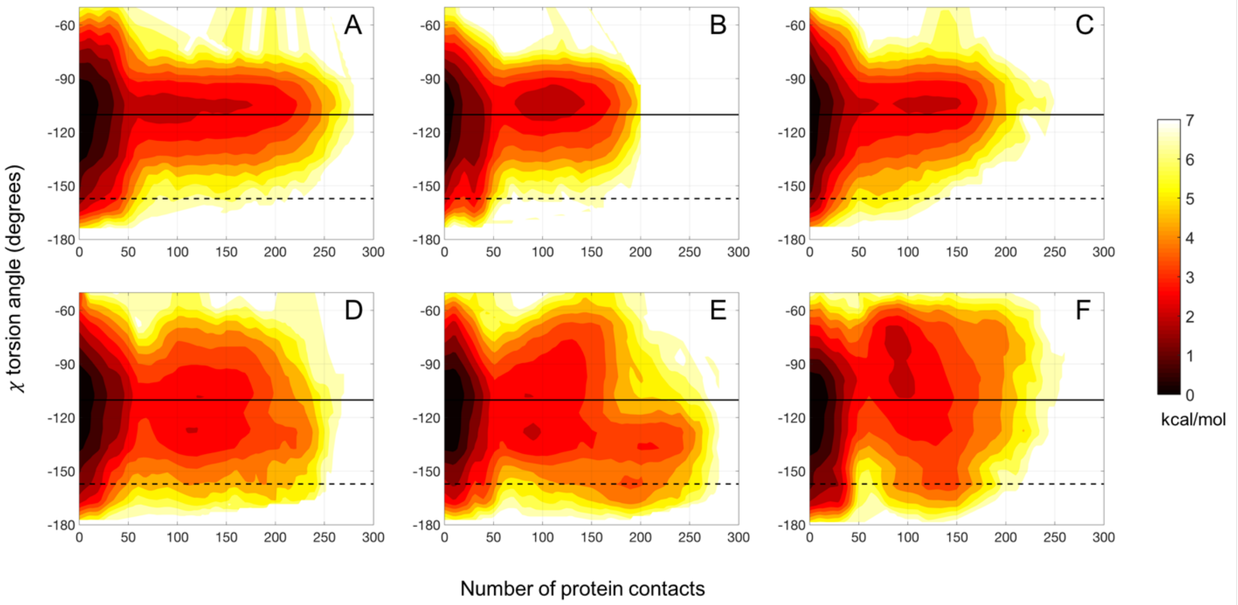

Figure S17. Potential of mean force (kcal/mol) as a function of χ backbone torsion angle and number of protein contacts for the Drew-Dickerson dodecamer at 20 % (A), 30 % (B), 40 % (C) protein concentrations, and for the GC-rich dodecamer at 20 % (D), 30 % (E), 40 % (F) protein concentrations. A contact is defined when the minimum distance between the heavy atoms of crowder proteins and DNA phosphate groups is less than 5 Å. Solid and dashed lines indicate the canonical B- and A-form values, respectively. See Table S5 for uncertainties.

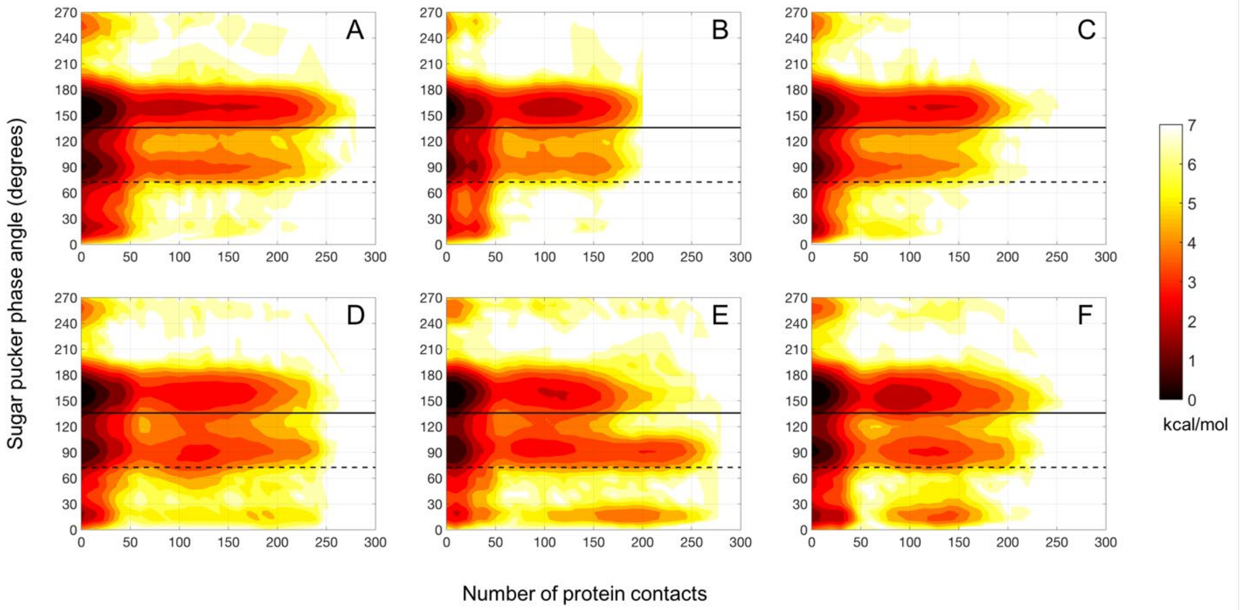

Figure S18. Potential of mean force (kcal/mol) as a function of sugar pucker phase angle and number of protein contacts for the Drew-Dickerson dodecamer at 20 % (A), 30 % (B), 40 % (C) protein concentrations, and for the GC-rich dodecamer at 20 % (D), 30 % (E), 40 % (F) protein concentrations. A contact is defined when the minimum distance between the heavy atoms of crowder proteins and DNA phosphate groups is less than 5 Å. Solid and dashed lines indicate the canonical B- and A-form values, respectively. See Table S5 for uncertainties.

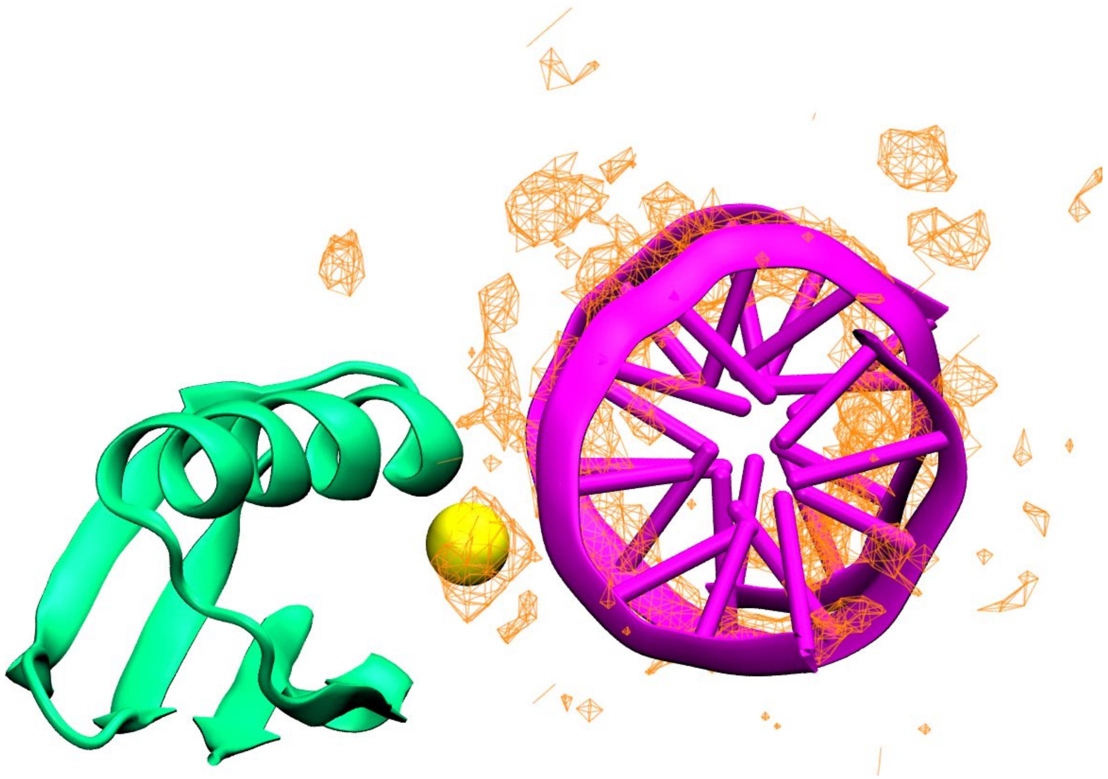

Figure S19. A snapshot showing a crowder protein interacting with the Drew-Dickerson DNA and orienting a sodium ion at the same time.
